# Recent Advances in Thermally Insulated Drilling Pipes: Materials, Design Strategies, and Future Directions

**DOI:** 10.3390/polym18081004

**Published:** 2026-04-21

**Authors:** Izaz Ali, Muhammud Arqam Khan, Yang Ding, Chaozheng Liu, Mei-Chun Li

**Affiliations:** 1 School of Petroleum Engineering, China University of Petroleum (East China), Qingdao 266580, China; izazali252@gmail.com (I.A.); arqamkhan@neduet.edu.pk (M.A.K.); yanding@upc.edu.cn (Y.D.); 2 Co-Innovation Center of Efficient Processing and Utilization of Forest Resources, College of Materials Science and Engineering, Nanjing Forestry University, Nanjing 210037, China; lczwood@njfu.edu.cn; 3 Department of Petroleum Engineering, NED University of Engineering & Technology, University Road, Karachi 75270, Pakistan; 4 Key Laboratory of Unconventional Oil & Gas Development, China University of Petroleum (East China), Ministry of Education, Qingdao 266580, China; 5 Shandong Key Laboratory of Oil and Gas Field Chemistry, China University of Petroleum (East China), Qingdao 266580, China

**Keywords:** thermal insulated drill pipe, polymer-based coating, silica aerogel, vacuum-insulated layer, phase change material

## Abstract

The increasing global demand for oil and gas, together with the depletion of shallow reservoirs, has driven exploration toward deep and ultra-deep formations characterized by high-temperature and high-pressure (HTHP) conditions. In such environments, conventional drill pipes often experience thermal stress, corrosion, and mechanical degradation, which can reduce drilling efficiency and compromise operational reliability. Thermal insulated drilling pipes (TIDPs) have therefore emerged as an effective solution to minimize heat transfer between drilling fluids and the surrounding formation. This review summarizes recent advances in TIDP materials, structural design strategies, fabrication technologies, and critical performance. Relevant studies were collected from major scientific databases, including Web of Science and Google Scholar, with a focus on insulation materials, coating technologies, and thermal management approaches used in drilling systems. The analysis indicates that advanced insulation systems, including polymer-based coatings, silica aerogels, vacuum-insulated layers, and phase-change materials, can significantly enhance thermal management in drilling operations. These technologies can reduce heat loss by approximately 40–60% (i.e., 400–600 W·m^−2^) and maintain drilling-fluid temperature differentials of 10–18 °C under HTHP conditions. In addition, fabrication techniques such as plasma spraying, composite fabrication, and additive manufacturing enable the development of multifunctional insulation systems with improved thermal, mechanical, and corrosion-resistant properties. Hybrid TIDP systems integrating nanocomposites and advanced polymers show strong potential for improving drilling safety and efficiency. However, challenges related to durability, scalability, and cost remain, highlighting the need for further research on multilayer insulation architectures and sustainable materials.

## 1. Introduction

Oil and gas play a central role in the global energy landscape and remain critical to the functioning of modern economies [1]. In recent years, driven by the growing global demand for energy and the gradual depletion of shallow, high-permeability resources, oil and gas development has undergone a significant shift from easily accessible conventional reservoirs to more technically challenging deep formations, where the depths often exceed 4500 m and downhole temperatures surpass 150 °C [2].

The high-temperature environments pose extreme drilling challenges, e.g., drilling fluid degradation, reduced tool reliability, and borehole instability, all of which increase the complexity and risk of drilling operation [3,4,5]. Particularly, elevated temperatures can degrade drilling fluid rheology, reduce the efficiency of cuttings transport, and destabilize boreholes [6]. Prolonged exposure to high temperatures causes a chemical breakdown of additives, phase separation, and filtration problems, undermining the ability of the fluid to maintain wellbore integrity [7]. Simultaneously, downhole tools and equipment, including rubber seals, measurement while drilling (MWD) and logging while drilling (LWD) sensors, and electronic components, are vulnerable to thermal degradation, leading to seal failure, battery malfunctions, and structural fatigue [8]. These failures not only increase operational risk but can also result in costly non-productive time (NPT) and compromised well integrity.

To mitigate these challenges, the use of thermal insulated drilling pipe (TIDP) has emerged as a promising solution [9,10,11]. TIDPs are specially designed drill strings incorporating thermal insulation materials between the inner and outer pipe walls to reduce heat exchange between the drilling fluid and the surrounding formation. The efficiency of TIDPs depends strongly on the selection of insulation material, bonding quality between the layers and the resistance to thermal degradation under high pressure, high temperature (HPHT) conditions. By maintaining lower fluid temperatures along the wellbore, TIDPs help preserve drilling fluid stability, extend tool lifespan, and improve overall thermal efficiency.

Recent advances in TIDP technology include the use of high-performance polymer coating, aerogels, and vacuum-insulated layer. In TIDPs, steel typically has a thermal conductivity of approximately 45 W·m^−1^·K^−1^, whereas polymeric insulation coatings exhibit much lower conductivities, generally ranging from 0.3 to 1.3 W·m^−1^·K^−1^, depending on their composition and structure. Silica aerogels possess exceptionally low thermal conductivities, typically in the range of 0.015–0.018 W·m^−1^·K^−1^. For vacuum insulation layers, thermal conductivities as low as ~0.004 W·m^−1^·K^−1^ can be achieved under high vacuum conditions (<1 mbar). However, under partially degraded vacuum conditions (approximately 200 mbar), the thermal conductivity may increase to around 0.008 W·m^−1^·K^−1^ [12,13,14]. These materials and structures provide enhanced thermal resistance while maintaining sufficient mechanical strength under HPHT conditions. For instance, high-performance polymer coatings, such as epoxy or polyurethane, are gaining importance in current TIDP designs. These coatings offer improved thermal insulation along with corrosion and abrasion resistance. Similarly, aerogels, such as silica aerogels, are attractive because of their extremely low thermal conductivity making them excellent insulating materials. Vacuum-insulated layers are another option, in which a vacuum gap suppresses convection and conduction to achieve thermal isolation. These are particularly effective in low-pressure applications.

Past literature that concerns thermal insulated drill pipes (TIDPs) is relatively limited and mostly incorporated in the context of a larger geothermal drilling or drilling-fluid thermal management investigation as opposed to a dedicated critical review ethos. To illustrate, the Handbook of Best Practices in Geothermal Drilling gives a description of insulated drill pipe (IDP) as a method of lowering heat conduction between drilling fluids and overheated formations mostly to safeguard the tools used in drilling geothermal wells [15]. The handbook, however, is primarily practical in nature, and mostly descriptive in its discussion, without being in a systematic examination of preparation approaches, thermo-hydraulic behavior and mechanical dependability of insulated drill pipe systems. Moreover, other recent review papers have also looked into temperature-control technologies of deep and ultra-deep drilling. These papers overview a range of cooling techniques of drilling fluids, such as the surface cooling techniques, optimization of the drilling-fluid features, and the application of low cooling-conductivity drilling tools [2,16]. Even though these reviews acknowledge that insulated drill pipe is one of the possible temperature-management options, they are oriented towards the general cooling technologies, but not towards TIDPs in particular. Thus, there are no detailed considerations of the routes of fabrication, structural integrity, and limitations of operations of TIDP. In addition, the recent reports on the drilling technologies of super hot geothermal systems have included the use of the insulated drill pipe with the reference to the technology readiness and engineering feasibility [17]. These reports emphasize the possible disadvantages of operation, including the rise in pipe weight, decrease in the inner diameter and rise in the pressure losses. However, they are aimed at mainly technology-gap discovery and implementation evaluation, but not a complete analysis of the principles of TIDP design and its functionality and fields of use. Thus, in contrast to the existing literature on the subject of the review where the majority of authors discuss the mechanisms of thermal insulation or overall temperature-control techniques, a more detailed assessment framework is created in the given study. Through a systematic study of the technique of preparation, the functional use, and a field application of TIDPs, this review is aiming to offer a better insight on its engineering viability and operational consideration in high temperature formation.

**Figure 1:**
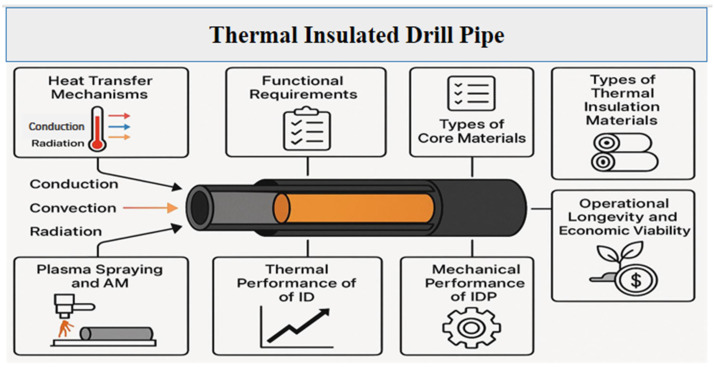
Illustration depicting a brief overview of this article.

This review systematically analyzes the preparation methods, functional performance, and field applications of TIDPs (Figure 1). Section 1 discusses the major heat transfer mechanisms, including conduction and convection, relevant to drilling operations. Section 2 and Section 3 focus on the key functional requirements for TIDPs and their fabrication techniques. This is followed by an evaluation of the critical performance indicators of TIDPs under HPHT conditions. Finally, the review outlines the future perspectives and challenges associated with the implementation of TIDPs in drilling operations. By incorporating interdisciplinary insights, this study promotes safer, more energy-efficient, and economically viable drilling operations, and serves as a valuable reference for researchers and engineers in the field of TIDPs.

To ensure transparency and minimize potential selection bias, a systematic literature search was conducted using Web of Science, Scopus, ScienceDirect, and Google Scholar. The search employed keywords such as “thermal insulated drill pipe,” “insulated tubing,” “HPHT drilling insulation,” “polymer coatings,” “aerogel insulation,” “phase change materials,” and “thermal management in drilling operations.” Peer-reviewed English-language articles published between 1960 and 2025 were included, as most recent technological developments in advanced insulation materials and HPHT drilling systems have been reported during this period. Titles, abstracts, and full texts of more than 425 initial records were screened. During the screening phase, 110 articles were excluded because they were unrelated to thermal insulated drilling pipes or focused on unrelated pipeline insulation systems. The remaining 325 articles were subjected to full-text evaluation. Among these, 68 studies were further excluded due to insufficient relevance, lack of technical data, or a focus on unrelated thermal management systems. Finally, 257 peer-reviewed articles were selected for detailed analysis and included in this review. These studies formed the basis for evaluating the materials, fabrication methods, thermal performance, mechanical properties, and corrosion resistance of thermally insulated drilling pipes.

## 2. Heat Transfer Mechanisms in Drilling Operations

**Figure 2:**
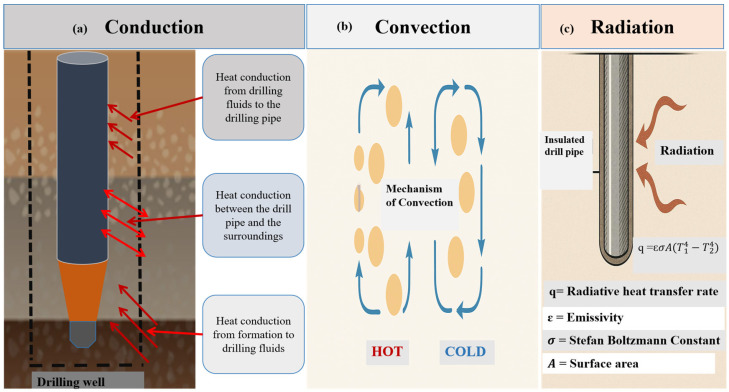
Illustration showing heat transfer mechanisms during drilling operations: (**a**) conduction, (**b**) convection, and (**c**) radiation.

Efficient thermal management is a critical aspect of modern drilling operations, particularly in high-temperature and ultra-deep wells. As drilling progresses into deeper formations with elevated geothermal gradients, the temperature difference between the wellbore and the circulating drilling fluid increases significantly. This temperature differential drives continuous heat exchange, which can adversely affect the performance of drilling fluids, downhole tools, and wellbore stability. Understanding the fundamental mechanisms by which heat is transferred within the wellbore environment is essential for designing effective insulation systems and optimizing thermal control strategies. Conduction, convection, and radiation are the primary modes of heat transfer in the drilling system and collectively determine the thermal profile along the drill string and annular space (Figure 2).

### 2.1. Conduction

Heat transfer by conduction occurs through direct molecular contact, such as in the metallic body of the drill pipe or the surrounding formation [18]. Heat flows from the high-temperature zone (i.e., the deep geothermal reservoir) to cooler regions (i.e., upper wellbore sections or surface equipment) via the drill pipe, which is typically made of high-thermal-conductivity steel (~40 to 50 W·m^−1^·K^−1^) [19]. A simplified schematic of heat conduction in drilling a well is shown in Figure 2a, in which primary heat conduction from the surrounding rock mass to the drilling fluid drives the overall heat flow into the wellbore. Particularly, there are three stages of conduction that occur during drilling operations: (1) heat conduction from the formation to the drilling fluid, (2) heat conduction from the drilling fluid to the drill pipe, and (3) heat conduction between the drill pipe and the surrounding environment [20].

In the first stage, the rate of heat transfer is strongly dependent on the thermal conductivity of the formation. Shale exhibits lower thermal conductivity, resulting in slower heat transfer, while sandstone has higher conductivity, allowing for faster heat exchange [21]. Yang et al. found that the accuracy of real-time downhole temperature forecasts is greatly increased when time-dependent drilling parameters and formation heterogeneity are included in transient finite element heat transfer models. Their research showed that temperature estimate, which is essential for safe and effective drilling operations, may include significant inaccuracies if these dynamic and geological aspects are ignored [22]. Continuous thermal profiles, as outlined by Khankishiyev et al. are obtained through advanced logging technologies such as distributed temperature sensing (DTS) [23]. Key influencing factors include formation lithology, porosity, and the thermal properties of drilling fluids, such as specific heat capacity. Additionally, the use of insulated casing in permafrost regions helps mitigate conductive heat loss and regulates heat transfer from the formation to the drilling fluids [24].

In the second stage, heat is transferred from the drilling fluid to the drill pipe through direct contact. The effectiveness of this transfer is influenced by fluid stagnation time and thermal diffusivity [25]. Nano fluids can enhance thermal dissipation by up to 20% through improved heat transfer characteristics [26]. Key factors affecting this stage include mud circulation rate, viscosity, pipe surface roughness, and the contact area. Therefore, optimizing mud rheology is essential to balance thermal conductivity and hydraulic efficiency [27].

In the third stage, heat conducts from the drill pipe to the surrounding environment, either into the annulus or the formation. In offshore or cold environments, conductive heat loss to cold seawater may lead to hydrate formation [28]. To counteract this, vacuum-insulated tubing (VIT) can reduce conductive losses by 80–90%, helping to maintain mud temperatures above hydrate formation thresholds [29]. Key parameters in this stage include the thermal conductivity of the annular fluid (e.g., oil-based and water-based mud) and the insulation quality of wellbore components. Deploying VIT is particularly effective in managing heat transfer in ultra-deepwater wells. Although this layered-resistance approach provides a useful approximation, it often overlooks interfacial thermal resistance between dissimilar materials, which can significantly affect overall insulation efficiency.

In multilayer TIDPs consisting of aerogel, polymer, and steel, the overall thermal resistance is approximately from 0.5 to 0.625 m^2^·K·W^−1^. Among the individual layers, the thermal resistance of aerogel, polymer, and steel is about 0.00018–0.00025, 0.025–0.035, and 0.50–0.065 m^2^·K·W^−1^, respectively. Accordingly, the contribution of steel is <1%, that of the polymer layer is <5%, while the aerogel layer contributes approximately 94% of the total thermal resistance [30]. Despite their significant thermal advantages, TIDPs introduce certain hydraulic limitations that must be considered during drilling operations. The multilayer insulation structure typically reduces the internal flow diameter of the drill pipe, which increases fluid velocity and frictional pressure losses for a given circulation rate. For example, a 10–15% reduction in internal diameter may increase frictional pressure losses by approximately 20–30%, depending on the flow regime and the properties of the drilling fluid [31,32]. This effect may lead to an increase in equivalent circulating density (ECD), particularly in deep or narrow well bores where hydraulic margins are already limited [32]. Higher ECD values can potentially increase the risk of lost circulation or wellbore instability. In addition, maintaining sufficient annular velocity for effective hole cleaning requires careful optimization of the mud circulation rate and rheological properties. Therefore, the implementation of TIDPs requires a balance between thermal retention benefits and hydraulic performance. Proper hydraulic modeling and operational planning are necessary to ensure that improvements in thermal insulation do not compromise drilling efficiency or wellbore stability.

### 2.2. Convection

Heat transfer via fluid movement in the annulus is known as convection (Figure 2b). After absorbing heat from the formation, hot drilling fluids circulating through the drill pipe ascend and transfer heat to the cooler surrounding formation or to the return fluids during circulation [33]. In geothermal wells, convective losses can reduce fluid temperature by up to 15% before the fluid reaches the surface, thereby lowering energy recovery efficiency [34]. Convective cooling in risers during deep-water oil drilling can promote hydrate formation, which is mitigated by using insulated risers or active heating systems [35]. Synthetic-based muds (SBMs), which possess higher heat capacity than water-based muds (WBMs), perform better under HPHT conditions [36].

Annular flow regimes are typically simulated using computational fluid dynamics (CFD) models to predict thermal hotspots and optimize mud velocity [37]. Modern vacuum-insulated drill pipes (VIDPs) help reduce convective losses by eliminating fluid contact within annular gaps [38]. A critical parameter influencing convective heat transfer is the Reynolds number (Re). For annular flow in drilling operations, the Reynolds number is typically on the order of 10^4^, which determines the flow regime: (1) laminar flow (Re < 2100), which results in poor convective heat transfer, and (2) turbulent flow (Re > 4000), which enhances fluid mixing and heat transfer [39]. Under turbulent conditions, the convective heat-transfer coefficient increases approximately with the fluid velocity raised to the power of 0.8:

h ∝ V^0.8^ (1)

where h is the heat-transfer coefficient and V is the fluid velocity. Consequently, doubling the mud velocity can increase the convective heat-transfer coefficient by approximately 70–75%. This relationship is consistent with classical turbulent heat-transfer correlations, such as the Dittus–Boelter equation [40,41]. Consequently, TIDP design often incorporates vacuum or low conductivity layers to suppress internal convective heat transfer and improve thermal retention.

### 2.3. Radiation

Although thermal radiation has historically been considered a minor heat transfer mechanism in drilling operations (Figure 2c), its significance is increasing in supercritical reservoirs and ultra-deep geothermal wells, where temperatures often exceed 350 °C [42]. Radiation involves the emission and absorption of electromagnetic waves, enabling heat transfer even through a vacuum or stagnant fluid. It is unlike conduction and convection, which require a material medium for heat transfer [43]. When convective cooling is limited and conductive pathways are insulated, radiative heat transfer becomes particularly important in confined annular spaces, double-walled drill pipes, or during periods of intermittent circulation. The Stefan–Boltzmann equation controls the rate of radiative heat transfer [44]:

(2) $$q=\varepsilon \cdot \sigma \cdot A\cdot (T_{1}^{4}-T_{2}^{4})$$

where q is the radiative heat transfer rate (W), ε is the surface emissivity (dimensionless, between 0 and 1), σ is the Stefan–Boltzmann constant (5.67 × 10^−8^ W/m^2^·K^4^), A is the surface area (m^2^), and $T_{1}$ and $T_{2}$ are the absolute temperatures (K) of the emitting and receiving surfaces, respectively. Using the Stefan–Boltzmann equation, and assuming an environment at 25 °C with a surface area of 1 m^2^, the radiative heat-transfer rate at 350 °C (623 K) is approximately 2430 W·m^−2^ for an emissivity of 0.3 and 810 W·m^−2^ for an emissivity of 0.1. At 450 °C (723 K), the corresponding values increase to about 4520 W·m^−2^ and 1500 W·m^−2^, respectively. These results demonstrate the strong dependence of radiative heat transfer on both temperature and surface emissivity.

Radiative heat flux can make a substantial contribution to the total heat load in high-temperature drilling environments, especially in HPHT and geothermal wells. Although the emissivity of metals like steel is relatively low (ε = 0.3–0.5), surface oxidation or degradation during drilling may increase emissivity, thereby intensifying radiative heat loss. In small, circular regions with restricted fluid movement, where natural convection is suppressed, radiation may account for over 20% of the total heat transfer, particularly when temperatures exceed 400 °C

[45].

To mitigate this, recent research has focused on applying low-emissivity surface coatings to the inner and outer layers of insulated drill pipes [46]. Materials such as aluminized polymer films, ceramic–metal (cermet) composites, and nano-enhanced reflective polymers are being developed to reflect heat and reduce net radiative flux. Among these, aluminized polyimide-siloxane coatings stand out due to their low emissivity values (as low as 0.1) and thermal stability up to 500 °C [47]. These coatings are lightweight, corrosion-resistant, and can be applied through various methods including spraying and thermal lamination. In a recent study, a 40% reduction in radiative heat loss in simulated geothermal well conditions was achieved using a multilayer polymer coating incorporating a boron nitride nano sheets [48]. The coating showed excellent adhesion to steel substrates, thermal stability across multiple heating cycles, and resistance to chemical attack by drilling fluids. Similarly, a vacuum-sputtered ceramic–polymer hybrid film was prepared, which retained both mechanical integrity and reflectivity, making it suitable for long-term deployment in deep drilling environments [49]. Beyond their radiative insulation properties, these advanced polymer coatings offer synergy with other thermal management strategies, such as conductive and convective barriers, enabling the development of multi-functional insulation systems. Their application is especially valuable in tool joints, transition zones between hot and cooled sections, and stagnant flow areas along the drill string. For this reason, low emissivity coatings are increasingly incorporated into advanced TIDP insulation systems to minimize radiative heat losses in high temperature wells.

### 2.4. Interplay of Mechanisms

All three heat transfer mechanisms, i.e., conduction, convection, and radiation, occur simultaneously in practice. Conductive losses through the pipe wall raise the temperature of the annular fluid, which in turn initiates convective currents that further transfer heat away. Additionally, radiant emissions from the pipe surface contribute to the overall thermal losses. This combined effect can be addressed by advanced insulation systems, such as aerogel-lined multilayer pipes, which incorporate low-conductivity materials to reduce conduction, vacuum gaps to impede convection, and reflective layers to block radiative heat transfer [50].

## 3. Functional Requirements of TIDPs

**Figure 3:**
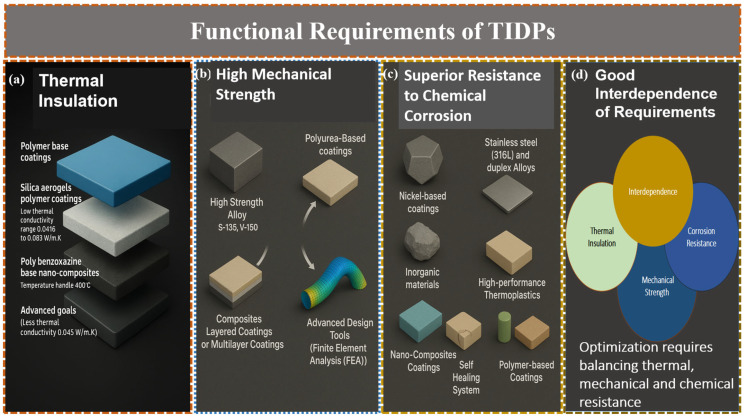
Functional Requirements of TIDPs: (**a**) effective thermal insulation, (**b**) superior mechanical properties, (**c**) high resistance to chemical corrosion, and (**d**) strong interdependence among these requirements.

TIDPs must balance multiple functional demands to perform reliably in harsh drilling environments. Key requirements include effective thermal insulation, superior mechanical properties, high resistance to chemical corrosion, and strong interdependence among these requirements (Figure 3).

### 3.1. Effective Thermal Insulation

Effective thermal insulation is essential in drilling operations to prevent excessive heat transfer between the drilling fluid inside drilling pipe and the surrounding formation or wellbore. This is particularly critical in high-temperature environments such as geothermal wells. Recent advancements in polymer coatings have significantly enhanced the thermal insulation performance of TIDPs [51]. One notable development is the use of silica aerogels encapsulated within polymer coatings. These coatings offer low thermal conductivity ranging from 0.0416 to 0.083 W·m^−1^·K^−1^, making them highly suitable for high-temperature applications [52]. The integration of aerogel-infused polymers into coating processes enhances both insulation and durability. This combined advantage is especially significant in geothermal operations, where pipelines endure both intense heat and pressure. Research shows that silica aerogel-polyimide composites can lower pipeline heat transfer to less than 0.035 W/m·K, improving thermal insulation efficiency while still being flexible [53]. Recent improvements include coatings made from polybenzoxazine-based nanocomposites, which can handle high temperatures up to 400 °C and resist damage from heat radiation. These coatings sustain performance under cyclic thermal stresses, diminishing the probability of fracture propagation or coating delamination. Collectively, these advancements ensure reduced radiative heat transmission while preserving the structural integrity of the TIDPs under harsh downhole conditions.

In geothermal and ultra-deep high-temperature drilling, the required thermal conductivity for effective insulation is usually less than 0.06 W·m^−1^·K^−1^, with advanced goals aiming for less than 0.045 W·m^−1^·K^−1^ to greatly lower radiative heat. This level can be attained by integrating silica aerogels with high-temperature polymers, like polyimides, polybenzoxazine, or epoxies. Adding materials like TiO_2_ and carbon black to these mixtures, along with special coatings that reflect heat, helps lower both heat transfer through conduction and radiation. Additionally, using layered or specially designed composite materials can simultaneously improve thermal insulation and mechanical strength, making sure they work well in high-pressure and high-temperature situations while keeping low heat transfer rates.

### 3.2. High Mechanical Strength Under Extreme Pressures and Temperatures

In HPHT environments (typically exceeding 175 °C and 10,000 psi), TIDPs must withstand substantial mechanical stresses, including axial loads, torsion, and collapse pressures encountered in deep wells. High-strength alloys such as S-135 and V-150, with yield strengths around 150,000 psi, have traditionally been used due to their excellent fatigue resistance [54]. However, the incorporation of thermal insulation layers, such as polymer coatings, presents challenges in maintaining structural integrity under these extreme conditions [55]. Recent advancements in polymer coatings have focused on enhancing mechanical strength without compromising thermal insulation. For instance, polyurea-based coatings have significantly increased the service life of polyethylene and polypropylene pipes, extending failure times by up to 200% under HPHT conditions as reported by Al Tamimi and Nelson in 2023 [56]. These coatings offer excellent elasticity and adhesion, which are critical for withstanding mechanical stresses in HPHT wells. Composite layered coatings have also been developed to provide both impact and heat resistance by combining materials such as oraganosilicon compounds with polyurethane interlayers. These multilayer systems exhibit superior impact strength, flexibility, and adhesion, surpassing conventional standards for corrosion protection in steel pipelines [57]. By efficiently distributing mechanical loads, such designs reduce the risk of delamination or mechanical failure. Additionally, Ahmed and Mohammed found that the incorporation of nanomaterials, such as TiO_2_/ZnO core–shell pigments, into polymer matrices can improve mechanical properties, including ductility, impact resistance, and hardness, thereby enhancing overall coating durability [58].

To further enhance the mechanical properties of TIDPs, advanced design tools such as finite element analysis (FEA) can be employed to optimize hybrid TIDP systems. These models, facilitate the evaluation of stress distribution and help identify potential failure zones, enabling the development of coatings that maintain structural integrity under HPHT conditions [59]. In summary, the integration of advanced polymer coatings into TIDPs has led to significant enhancements in mechanical strength and durability under HPHT conditions. These improvements are critical for ensuring the efficiency and safety of drilling operations in challenging HPHT environments.

### 3.3. Superior Resistance to Chemical Corrosion

Corrosion resistance is critical in drilling operations, particularly because drilling fluids often contain corrosive agents such as carbon dioxide (CO_2_), hydrogen sulfide (H_2_S), and high-salinity brines. Traditional materials like nickel-based coatings and stainless steels, such as 316 L and duplex alloys, have been widely used due to their resistance to pitting and sulfide stress cracking [60]. To further enhance the resistance to chemical corrosion, several strategies, including the use of inorganic materials, high-performance thermoplastics, nano composite coatings, self-healing systems, and bio-based polymers, can be applied. For instance, Hegazy et al. found that, in offshore drilling environments, TIDPs with Al_2_O_3_ plasma-sprayed coatings show a decrease in corrosion rates when compared to uncoated pipes [61]. Furthermore, recent advancements in polymer-based coatings have significantly enhanced corrosion resistance in drilling applications. High-performance thermoplastics like Poly Ether Ether Ketone (PEEK) are well-suited for harsh downhole environments due to their exceptional chemical inertness and thermal stability up to 250 °C. PEEK-based composites have been successfully employed in components such as gaskets, liners, and seals, thanks to their resistance to aggressive chemicals and high-pressure conditions [62]. In addition, progress in polymer coatings has led to the development of nanocomposite materials. For example, hybrid coatings incorporating graphene into a polyetherimide (PEI) matrix have shown substantial improvements in corrosion resistance. These coatings enhance barrier properties and reduce corrosion rates by creating a tortuous path that impedes the penetration of corrosive agents [63]. Furthermore, self-healing polymer coatings have also emerged as a promising solution for maintaining long-term corrosion resistance. These systems contain micro-capsules filled with healing agents that are released upon mechanical damage, effectively repairing the protective layer and delaying corrosion onset [64]. Additionally, research has explored the development of environmentally friendly and sustainable coatings by incorporating natural polymers such as chitosan into epoxy matrices. For instance, Esmailzadeh et al. found that chitosan-enhanced coatings offer excellent film-forming ability, corrosion resistance, and antimicrobial properties, making them suitable for oil and gas applications [65]. These innovations contribute to increased durability and reliability of drilling equipment in corrosive environments.

### 3.4. Good Interdependence of Requirements

The development of TIDPs presents significant technical challenges due to the interdependence of thermal insulation, mechanical strength, and corrosion resistance. Enhancing one property often affects the others, necessitating integrated material solutions [66]. For instance, ceramic materials offer excellent thermal insulation but are inherently brittle and susceptible to mechanical failure under the dynamic stresses encountered in drilling operations.

To address this issue, hybrid materials such as carbon fiber-reinforced carbon aerogels (C/CAs) have been developed. These composites demonstrate low thermal conductivity (as low as 0.027 W·m^−1^·K^−1^) along with improved mechanical properties, including compressive strengths up to 2.14 MPa and flexural strengths of up to 3.62 MPa, depending on the carbonization temperature [67]. Such materials maintain structural integrity while delivering effective thermal insulation. Polymer-based coatings have also been enhanced with inorganic nanofillers to improve corrosion resistance and barrier performance. For example, Abdus samad et al., incorporated silica (SiO_2_) nanoparticles into epoxy matrices that resulted in increased hydrophobicity and reduced porosity, thereby improving resistance to corrosive agents [68]. However, the addition of these fillers requires careful control, as excessive loading can lead to agglomeration and reduced protective efficiency. Moreover, reliable adhesion between corrosion-resistant coatings and thermally insulating substrates is critical, especially under thermal cycling conditions [69]. Mismatches in thermal expansion coefficients between different material layers can result in delamination [70]. To mitigate this, composite coatings that integrate both thermal insulation and corrosion protection are being actively explored. Systems that combine polymer matrices with ceramic or metallic particles offer a promising balance of properties, ensuring durability and performance under harsh drilling conditions [71]. The formulation of TIDPs therefore requires a holistic approach that considers the interactions among thermal insulation, mechanical robustness, and corrosion resistance [72]. Advances in composite materials and nanotechnology are enabling the development of multifunctional coatings and structures that meet these complex performance requirements [73].

Thermal insulation, mechanical robustness, and chemical stability are three multifunctional requirements that must be carefully considered when designing TIDPs. The core material, insulating layers, and protective coatings work in concert to produce each property rather than achieving them separately. To guarantee optimum performance under HPHT conditions, material science and mechanical engineering principles must be integrated into the preparation and structural design of TIDPs. Therefore, the selection of core materials, insulation layers, and fabrication techniques that together allow TIDPs to meet the previously identified functional requirements is described in the following section.

## 4. Preparation of TIDPs

### 4.1. Selection of Core Materials

The TIDPs must be fabricated from core materials capable of withstanding HPHT conditions, as drilling in such environments imposes significant mechanical and chemical demands on the drill string. These materials must demonstrate high mechanical strength, corrosion resistance, and thermal stability to maintain operational integrity and safety. Table 1 summarizes the advantages, disadvantages, and future prospects of these core materials including high-strength low-alloy (HSLA), corrosion-resistant alloys (CRAs), carbon fiber reinforced polymers (CFRPs), and metal matrix composites (MMCs).

**Figure 4:**
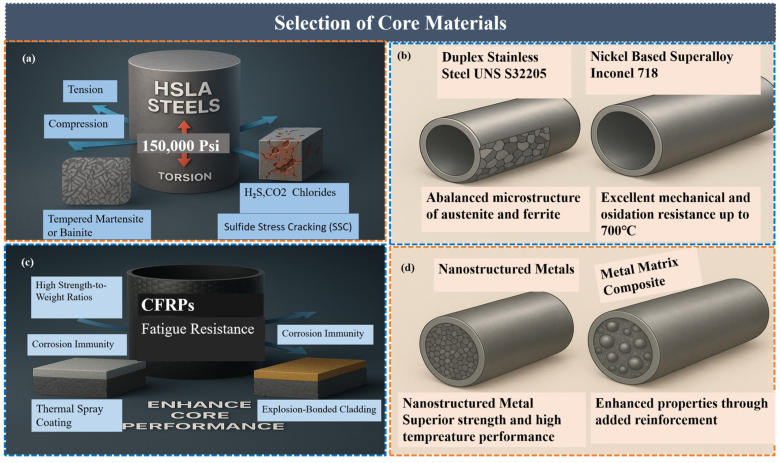
Selection of core materials: (**a**) HSLA Steels, (**b**) CRAs, (**c**) CFRPs, and (**d**) MMCs.

HSLA steels, such as API grades S-135 and V-150, exhibit yield strengths of approximately 150,000 psi and possess excellent tensile properties combined with good fatigue resistance [54]. HSLA steels perform robustly under axial tension, compression, and torsion conditions commonly encountered in directional drilling and extended-reach applications [74]. In TIDPs, the insulation layers are generally non-load-bearing, whereas the inner and outer HSLA steel tubes remain the principal structural components (Figure 4a). Nevertheless, the addition of insulation may modify the cross-sectional geometry and slightly affect the collapse resistance and torsional capacity. Therefore, these effects should be carefully assessed for each specific pipe design [41,75]. In environments containing hydrogen sulfide (H_2_S), carbon dioxide (CO_2_), chlorides, and other aggressive agents, conventional steels may suffer from sulfide stress cracking (SSC), pitting, and crevice corrosion, as reported by Liu et al. [76]. Furthermore, the high strength and toughness of their micro structures, typically tempered martensite or bainite, plays a key role in resisting these adverse effects under the cyclic loading conditions prevalent during rotary drilling operations [77]. To further minimize such risks, CRAs are increasingly being used in TIDP cores. Duplex stainless steels, such as UNS S32205 (commonly known as 2205), exhibit a balanced microstructure of austenite and ferrite, providing enhanced strength and localized corrosion resistance to meet demanding requirements [78]. Additionally, nickel-based super alloys such as Inconel 718 offer excellent mechanical and oxidation resistance up to approximately 700 °C, making them suitable for sour and HPHT well environments (Figure 4b). These alloys are particularly applied in critical pipe sections exposed to highly aggressive fluids [79].

Composite core materials, especially CFRPs, offer high strength-to-weight ratios, fatigue resistance, and corrosion immunity [80]. While CFRPs offer advantages in reducing overall pipe weight and improving handling, their relatively limited thermal stability (up to 200 °C) restricts their application in deeper HPHT wells [81]. Furthermore, sustained high-temperature exposure can lead to matrix softening and interfacial debonding [82]. Although hybrid designs incorporating metallic liners or thermal barriers have been proposed to broaden the applicability of CFRPs in thermally demanding environments [83], emerging research now focuses on advanced coatings and cladding techniques to enhance core performance without complete replacement. Thermal spray coatings using ceramic- or carbide-based materials can improve both wear and oxidation resistance [84], while explosion-bonded cladding enables the application of corrosion-resistant alloys onto carbon steel substrates to achieve a balance between mechanical strength and corrosion protection [85] (Figure 4c).

Furthermore, nanostructured metals and MMCs are being explored for future TIDP development due to their superior strength and high-temperature performance [86]. As drilling environments become increasingly deeper and more hostile, continuous advancements in metallurgical design, composite technologies, and protective laminating systems are essential [87] (Figure 4d). Future development efforts are expected to focus on hybrid and intelligent materials capable of adapting to dynamic downhole conditions.

**Table 1:** Advantages, Disadvantages, and Future prospects of core materials.

Material	Advantages	Disadvantages	Future Perspectives	References
HSLA Steels (e.g., API S-135, V-150)	High yield strength (~150,000 psi); excellent tensile, fatigue, and torsional performance; good toughness; cost-effective	Susceptible to SSC, pitting, and hydrogen embrittlement in severe H_2_S/CO_2_/chloride environments; inferior corrosion resistance to stainless/nickel alloys	Micro alloying and thermo mechanical processing for SSC resistance; advanced coatings; hybrid designs with corrosion-resistant cladding	[54,74,76,77]
CRAs (Duplex SS 2205, Inconel 718)	Superior corrosion resistance to H_2_S/CO_2_/chlorides; duplex offers balanced strength/pitting resistance; nickel alloys retain strength to ~700 °C	High cost; heavier than composites; duplex steels may lose toughness/corrosion resistance > 315 °C	Cost-optimized duplex/super-duplex grades; improved welding; explosion-bonded CRA cladding	[78,79]
CFRPs	High strength-to-weight ratio; corrosion immune; excellent fatigue resistance	Thermal stability limit (~200 °C); matrix degradation/fiber–matrix debonding; difficult damage detection and repair	Metallic liners/thermal barriers; high-temp resins (>250 °C); smart composites with embedded sensors	[80,81,82,83]
Nanostructured Metals and MMCs	Exceptional strength/hardness; high-temp performance with tailored matrices; potential wear/oxidation resistance	High manufacturing cost; limited field data; joining/repair challenges	Scalable manufacturing (powder metallurgy, additive manufacturing); multifunctional MMCs; adaptive “smart” drill pipe cores	[84,85,86,87]

### 4.2. Selection of Insulation Layers

The majority of insulation layers used in TIDPs are selected based on their thermal conductivity, temperature tolerance, and compatibility with aggressive drilling fluids encountered in deep and high-pressure environments [88]. The insulation layers must reduce heat transfer while withstanding extreme conditions such as high temperatures, high pressures, and exposure to corrosive substances. Currently, the insulation layers employed include polymer coatings, aerogels, vacuum-insulated layers, and phase change materials. The advantages, disadvantages, and future prospects of these insulation layers are summarized in Table 2.

**Figure 5:**
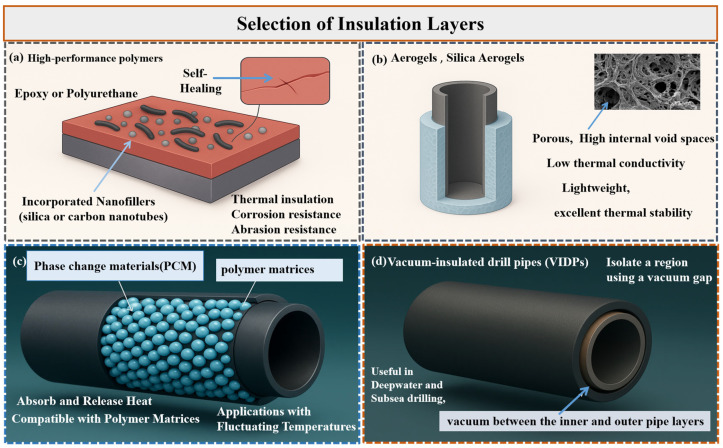
Selection of insulation layers: (**a**) High-performance polymer coatings, (**b**) Aerogels, (**c**) Phase Change Materials (PCMs), (**d**) Vacuum-Insulated Layers (VIDPs).

High-performance polymer coatings, such as those based on epoxy or polyurethane, are gaining importance in current TIDP designs, as shown in Figure 5a. These coatings offer improved thermal insulation along with corrosion and abrasion resistance [89]. They are designed to withstand the high mechanical stresses associated with drilling operations due to their flexibility and strong adhesion to steel or composite substrates [90]. Recent advancements have incorporated nanofillers, such as silica or carbon nanotubes, into these coatings to enhance mechanical strength [91]. Some polymer coatings are even engineered with self-healing properties, allowing them to recover from micro-cracks and extend service life under harsh conditions. These developments are particularly relevant for deepwater drilling, where strength, corrosion resistance, and thermal insulation are all critical [92].

For geothermal and HPHT wells, aerogels, such as silica aerogels, are attractive due to their extremely low thermal conductivity (approximately 0.015 W·m^−1^·K^−1^), making them excellent insulating materials [93]. These materials effectively reduce both conductive and radiative heat transfer, making them suitable for high-temperature applications exceeding 300 °C. Additionally, aerogels are lightweight, which reduces the overall weight of the drill string, an important factor in deep and ultra-deep well operations [94]. Aerogels also exhibit excellent thermal stability and maintain their insulating properties under extreme conditions. Studies have demonstrated their resilience in geothermal wells, where thermal cycling and corrosive fluids pose significant challenges [95] (Figure 5b). However, a known limitation is their fragility under mechanical stress and pressure, which recent developments in aerogel composites have sought to address [96].

Phase change materials (PCMs), such as paraffin compounds, are also being used in TIDP insulation layers due to their ability to absorb and release significant amounts of heat during phase transitions, as shown in Figure 5c. These materials are effective in applications with fluctuating temperatures, where they stabilize the system by absorbing excess heat during temperature spikes and releasing it when temperatures drop [97]. There is growing interest in integrating PCMs into advanced polymer coatings to provide both thermal insulation and temperature regulation in a single system. Recent studies have explored encapsulating PCMs within polymer matrices to enhance thermal energy storage capacity, mechanical strength, and durability. Some research has successfully developed shape-stable composites by encapsulating PCMs within particulate polymer networks, thereby combining latent heat storage with structural functionality [98]. Heat regulation can also be localized to specific critical areas of the pipe, such as joints or zones exposed to the highest temperatures, by embedding PCMs into coatings. Continued research is focused on developing PCMs that are stable at higher temperatures and compatible with high-performance polymer matrices [2].

Another innovative approach involves vacuum-insulated layers, which thermally isolate a region using a vacuum gap to suppress convection and conduction (Figure 5d). In low-pressure applications (below 0.001 atm), thermal conductivities as low as 0.008 W·m^−1^·K^−1^ have been achieved [99]. This technology is especially useful in deepwater and subsea drilling, where thermal stability is vital for maintaining wellbore integrity and tool performance [100]. Vacuum-insulated drill pipes (VIDPs) achieve this by incorporating a vacuum between the inner and outer pipe layers, significantly minimizing heat loss to the surrounding environment [101]. Recent research on VIDPs has focused on optimizing material selection for the vacuum chamber and enhancing the durability and integrity of seals to maintain the vacuum during drilling. One challenge is ensuring long-term seal stability under high-pressure conditions.

**Table 2:** Advantages, Disadvantages, and Future prospects of insulation layers.

Material/Insulation Layer	Advantages	Disadvantages	Future Perspectives	References
High-Performance Polymer Coatings	Thermal insulation with corrosion and abrasion resistance; flexibility and strong adhesion; nanofillers enhance strength; self-healing variants extend service life	Lower insulation than aerogels/vacuum; chemical degradation in aggressive fluids; self-healing limited under extreme HPHT	Integration with PCMs for combined insulation and heat regulation; advanced nanocomposites for higher temperature tolerance; smart coatings for real-time damage detection	[89,90,91,92]
Aerogels (Silica Aerogels)	Extremely low thermal conductivity (~0.015 W/m·K); reduces conductive and radiative heat transfer; lightweight; thermally stable > 300 °C	Fragile under mechanical stress and pressure; higher production and integration costs	Composite aerogels with fibers/polymers for toughness; functionalized aerogels resistant to chemical degradation; cost-effective large-scale manufacturing for deepwater and HPH	[93,94,95,96]
Phase Change Materials (PCMs)	Thermal regulation via heat absorption/release; ideal for fluctuating temperatures; can be integrated into polymer coatings; shape-stable composites add structural functionality	Limited temperature range; leakage risk; lower insulation than aerogels/vacuum	High-temperature-stable PCMs for HPHT wells; localized heat regulation at critical pipe sections; micro encapsulation for durability and coating integration	[2,97,98]
Vacuum-Insulated Layers (VIDPs)	Very low thermal conductivity (~0.008 W/m·K at <0.001 atm); excellent for deepwater and subsea; maintains wellbore integrity	Maintaining vacuum under HPHT challenging; high manufacturing complexity and cost; difficult repair if vacuum compromised	Advanced sealing materials for long-term vacuum stability; multilayer insulation integration; lightweight composite vacuum chambers	[99,100,101]

## 5. Fabrication Techniques

Polymer/composite coating, plasma spraying, and additive manufacturing (AM) techniques are increasingly used in the fabrication of TIDPs due to their ability to apply high-performance thermal barrier coatings. Polymer/composite coatings are a thin layer of polymer or a combination of polymer and other materials (composites) are deposited on a substrate to enhance material performance. These coatings have broad scopes of application including corrosion, abrasion, and chemical protection, as well as improved surface characteristics. Plasma spraying enables the deposition of durable ceramic or metallic coatings without compromising the structural integrity of the pipe. AM processes, such as directed energy deposition, can produce drill pipes with integrated insulating features, such as layered materials or lattice cores, which enhance both mechanical strength and thermal resistance. Although plasma spraying and AM methods still face challenges related to cost and scalability, recent advancements in automation and materials science are improving their feasibility for field deployment.

### 5.1. Polymer/Composites Coating

TIDPs and related applications exhibit significant variations in both coating processes and resulting performance. Therefore, various polymer coating techniques are first discussed. In dip coating, substrates are immersed in and withdrawn from a liquid, enabling cost-effective and uniform coverage of complex geometries [102]. In contrast, spray coating relies on atomization of the coating material, allowing rapid coverage of large areas but with material losses due to overspray [103]. Blade coating and spin coating produce highly uniform thin films and are mainly suitable for planar or small substrates, with spin coating offering particularly high precision at nanoscale thicknesses. Thermal spraying is used to deposit molten materials, forming thick, hard, and wear- and corrosion-resistant coatings [104], whereas plasma polymerization generates thin, cross-linked polymer films with strong adhesion and excellent chemical resistance [105]. Composite coatings are prepared from mixtures of powders and polymers, combining functional fillers, such as ceramics or carbon nanotubes, with polymer binders to provide flexibility, adhesion, and tailored thermal or mechanical properties. These coatings have been applied as insulation layers in drilling equipment [106]. Ceramic or hybrid coatings produced via sol–gel methods exhibit high thermal stability, although careful drying is required to avoid defects [107].

**Figure 6:**
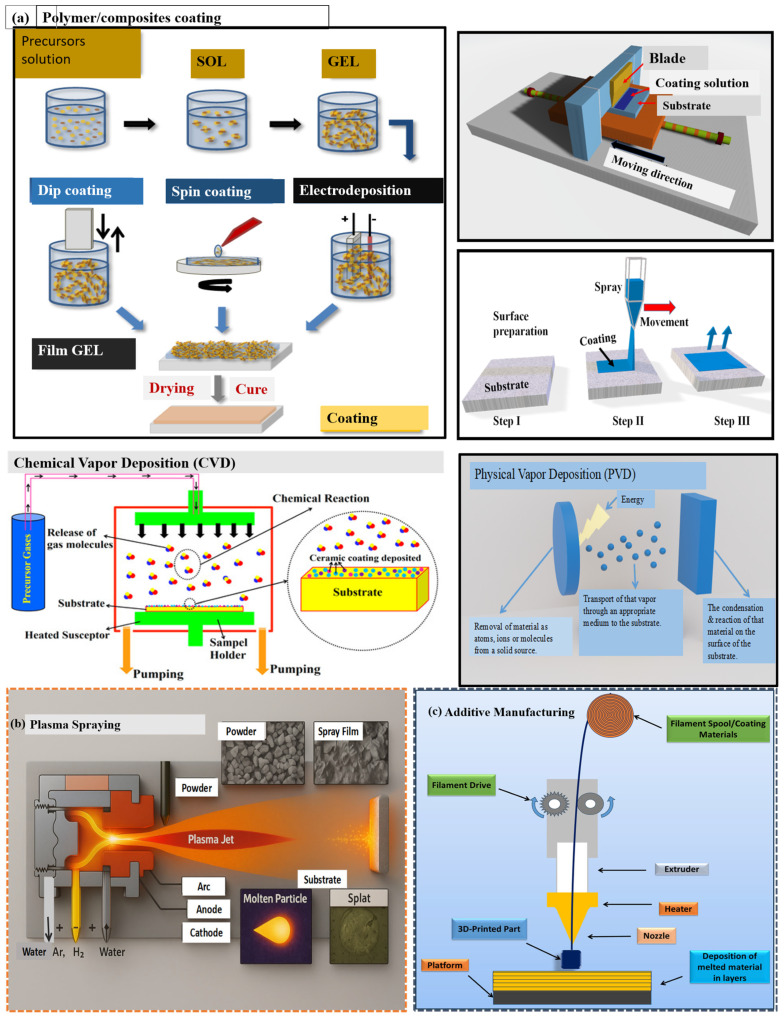
Fabrication techniques of TIDPs: (**a**) polymer/composite coating, (**b**) Plasma spraying, and (**c**) additive manufacturing.

Moreover, electrodeposition enables metals, conductive polymers, or composite coatings to be deposited onto conductive substrates using an electric current, allowing controlled and uniform coatings on complex geometries for corrosion and wear protection [108]. Chemical vapor deposition (CVD) operates in the vapor phase, where a heated substrate induces decomposition or reaction of volatile precursor gases to form dense, uniform coatings with high adhesion and purity. CVD has been widely applied in TIDPs using ceramic, diamond-like carbon, and polymeric films due to their excellent thermal, corrosion, and wear resistance [109]. Physical vapor deposition (PVD) involves the evaporation of precursor materials in a vacuum chamber, followed by condensation of vaporized atoms onto a substrate to form metal or metal-oxide nanoparticles. This process offers high purity, well-controlled crystallinity, and excellent control over particle size and homogeneity [110]. Figure 6a presents a summary of the major polymer and composite coating methods reported in the literature. Among these techniques, plasma polymerization and CVD demonstrate superior adhesion and chemical stability, making them particularly suitable for high-temperature TIDP applications.

### 5.2. Plasma Spraying

Thermal spray coating, including plasma spraying, is a technique that utilizes a high-velocity jet of ionized gases to melt and propel coating materials onto a substrate. This method enables the deposition of a wide variety of materials, such as ceramics and metals, onto surfaces like drilling pipes, forming protective coatings [111]. Plasma spraying is highly versatile, capable of depositing metals, ceramics, and polymers, which are essential for enhancing the performance of components operating in extreme environments, such as those encountered in oil and gas drilling [112]. This process offers a fast and economical way to manufacture durable coatings with superior mechanical, thermal, and corrosion resistance, making it particularly suitable for drilling pipes exposed to harsh downhole conditions, as shown in Figure 6b. One of the major applications of plasma spraying in the oil and gas industry is for corrosion and thermal protection of drilling pipes [113].

The most commonly used materials are alumina (Al_2_O_3_) and zirconia (ZrO_2_), which offer protection against corrosion and high-temperature oxidation [114]. Composite coatings such as alumina-impregnated carbide, further enhance wear resistance and corrosion protection in highly aggressive drilling environments, including those with saline or acidic fluids [115]. Zirconia-based thermal barrier coatings are especially valued for their low thermal conductivity and high-temperature tolerance [116]. In HPHT conditions, where extreme temperatures degrade the base materials, these coatings serve as effective thermal insulators and prevent substrate degradation from prolonged heat exposure [117]. For applications requiring high mechanical strength, plasma-sprayed coatings provide excellent bond strength, often exceeding 50 MPa. This makes the process well-suited for HPHT environments. Additionally, plasma spraying offers rapid deposition, allowing coatings to be applied quickly over large areas, an advantage for industrial operations where uptime and efficiency are critical [118]. Moreover, plasma spraying is compatible with complex geometries, enabling effective coating of irregularly shaped parts and drill pipe sections with intricate features [119]. This adaptability is crucial to ensuring comprehensive protection across all segments of a drill pipe, especially in offshore or deepwater drilling applications. Plasma spraying can also be applied to a range of substrates, including steels, composites, and some non-metallic surfaces, broadening its applicability across drilling equipment [120].

Despite its advantages, plasma spraying has some limitations, most notably the inherent porosity of the deposited coatings, which typically ranges from 5 to 15%. This porosity compromises the protective barrier by allowing corrosive fluids to penetrate and reach the substrate, thereby accelerating degradation [121]. Fluid ingress through pores can lead to rapid corrosion, even if the onset is slightly delayed compared to direct corrosion. This issue is especially problematic in fluids that promote polarization corrosion [122]. Therefore, post-spray treatments like sealing or densification have become integral to modern plasma-spray workflows.

Techniques like hot isostatic pressing (HIP) or the application of additional sealing layers are commonly employed to improve coating integrity. Furthermore, process optimization, such as refining spray parameters and using higher-purity feed stock, can reduce porosity and minimize stress-induced defects, as reported by Dehghani et al. [123]. Another strategy involves the use of composite coatings, which combine the advantages of ceramics and metals. These composites reduce thermal mismatch and enhance mechanical performance [124]. Continued advancements in spray parameter control, such as adjustments to temperature, standoff distance, and feed stock composition, have significantly improved coating quality and reduced defect rates [125]. Recent innovations include hybrid coatings that integrate plasma-sprayed ceramics with polymer matrices. These coatings offer enhanced flexibility, reduced brittleness, and improved adhesion. Polymers such as epoxy, polyurethane, and polyimide are commonly used to complement ceramic coatings and further enhance corrosion resistance [126]. Such hybrid systems combine the best properties of both materials, helping to overcome the inherent brittleness of ceramic coatings.

Today, plasma spraying remains one of the most effective methods for applying durable protective coatings to drilling pipes and other key components in oil and gas exploration. With the growing need for performance in HPHT and deepwater environments, the ability to apply corrosion-resistant and thermally insulating coatings has become essential. However, careful management of porosity and thermal stresses is necessary to ensure long-term coating performance. Future drilling operations are expected to benefit from continued innovations in process optimization, composite material development, and hybrid coating technologies.

### 5.3. Additive Manufacturing

Commonly referred to as additive manufacturing (AM) or 3D printing, this technology is transformative for the oil and gas industry, particularly in the fabrication of thermally insulated drilling pipes (TIDPs). Numerous examples demonstrate how AM is used to create complex, customized components with optimized material properties by precisely depositing materials in a layer-by-layer manner [127] (Figure 6c). This design flexibility is particularly beneficial for tailoring thermal gradients along multi-zone wells. AM offers significant potential for innovation in the manufacturing of drilling pipes, especially through the use of materials capable of delivering superior thermal and mechanical performance in extreme downhole environments. It enables the development of more efficient and durable drilling technologies by allowing the fabrication of structures with tailored geometries and material compositions [128].

One of the most relevant AM technologies for addressing thermal challenges in drilling pipes is Directed Energy Deposition (DED). DED is a process in which 3D printing is carried out by depositing materials layer by layer using a focused energy source, typically lasers, electron beams, or plasma arcs [129]. During this process, micron-scale metal-ceramic hybrid layers can be printed onto the surface of the pipe, combining the structural strength of steel with the thermal insulation properties of ceramics [130].

Additive manufacturing allows for the customization of insulation layers in TIDPs. This includes the ability to incorporate diverse material combinations and thicknesses along different pipe sections, achieving high precision and optimized thermal performance across varying wellbore zones [131,132]. This flexibility is especially beneficial in complex drilling operations, where temperature gradients can vary significantly along the pipe length. Numerical heat-transfer simulations indicate that the axial temperature gradient of conventionally coated drill pipes is approximately 3–5 °C·m^−1^, whereas additive-manufactured TIDPs exhibit a lower axial temperature gradient of about 1–2 °C·m^−1^, owing to their multilayer insulation structures and reduced radial heat loss [133]. Additionally, additive manufacturing reduces material waste by depositing only the required amounts of material, enhancing sustainability and lowering production costs [134].

Despite these advantages, several challenges hinder the widespread adoption of AM for TIDP production. One major barrier is the high cost of AM equipment, especially systems like DED and SLS, which are expensive and not yet cost-effective for high-volume production. The need for specialized machines capable of handling the high temperatures, tight tolerances, and complex geometries typical of TIDP components further increases initial capital investment. This is particularly burdensome for companies operating on narrow profit margins. Furthermore, while AM allows for customization and rapid prototyping, it currently lacks the scalability required for mass production. The printing speed is slower than traditional manufacturing methods, and the technology may not yet be robust enough to support high-throughput production of drilling pipes without significant cost or time penalties. These scalability limitations restrict the full integration of AM into the oil and gas supply chain, particularly where demand for drilling equipment is high [135,136].

Nevertheless, recent developments show promise. The emergence of advanced polymer materials with enhanced thermal and pressure resistance has led to the creation of polymer-based coatings suitable for AM processes. When these polymers are combined with ceramic fillers or aerogels, the resulting composites offer improved insulation while maintaining structural strength. Researchers are also working to improve the efficiency and reduce the cost of AM by developing novel materials, increasing printing speed, and simplifying the process. Some studies focus on multi-material 3D printing systems capable of simultaneously printing metals, ceramics, and polymers. This capability provides even greater design flexibility and performance tuning for drilling pipes with integrated insulation layers [137,138].

In summary, AM represents a revolutionary advancement in the production of thermally insulated drilling pipes. It offers numerous benefits, including customizable insulation thicknesses, reduced material waste, and the ability to produce complex geometries. The integration of hybrid materials, such as metal-ceramic coatings and aerogel composites, has significantly improved the thermal and mechanical properties of TIDPs. However, challenges such as high equipment costs and limited scalability must be addressed. Continued research into material development, process optimization, and scalability will be key to unlocking the full potential of AM for TIDP applications in the oil and gas industry.

## 6. Critical Performance of TIDPs

### 6.1. Thermal Performance

The thermal efficacy of TIDPs refers to their ability to reduce heat exchange between the drilling fluid and the surrounding formation in geothermal or high-pressure high-temperature (HPHT) wells. The primary methodologies used to evaluate insulation effectiveness include thermal conductivity, bottom-hole circulating temperature (BHCT), and heat retention efficiency [139]. Among these, thermal conductivity is the most critical parameter, as it directly determines a material’s ability to transfer heat; lower thermal conductivity corresponds to better insulating performance [140]. Thermal conductivity is commonly measured using techniques such as the guarded hot-plate or transient hot-wire methods. In contrast, BHCT reduction and overall heat retention within the wellbore under operating conditions are typically evaluated through comprehensive circulation experiments and numerical simulations [141,142]. In recent years, polymer-coating-based, aerogel-based, vacuum-based, and phase-change-material (PCM)-based TIDPs have been developed and applied as thermal insulation systems. The following subsections comprehensively discuss the thermal insulation performance of these TIDP technologies.

Polymer coatings are widely used in TIDPs due to their ease of processing, strong adhesion to substrates, and good thermal and chemical resistance. Commonly employed polymers include epoxy resins, polyurethanes, and high-temperature polyimides, which are applied as insulating barrier layers either on the inner or outer surfaces of the drill pipe. However, their relatively high thermal conductivity, typically around 0.2 W/m·K, limits their effectiveness under extremely high-temperature and high-intensity drilling conditions [143]. To enhance thermal performance, polymer coatings are often combined with inorganic fillers such as silica or alumina, which improve thermal resistance and stability while maintaining adequate mechanical strength [144]. In addition, the incorporation of reflective metallic interlayers, such as aluminum coatings, in conjunction with polymer layers has been shown to effectively reduce radiative heat loss, particularly in gas-rich drilling environments [145]. For example, ethylene tetrafluoroethylene (ETFE) powder coatings have been demonstrated to significantly improve the thermal insulation performance of steel drill pipe analogs. Coated samples with thicknesses ranging from 40 to 80 mil exhibited thermal conductivity values 45–60% lower than those of uncoated steel. Correspondingly, temperature differentials increased to 7–12 °C, compared to approximately 5 °C for uncoated steel. Although the ideal relationship between coating thickness and thermal conductivity remains unclear, ETFE coatings exhibited excellent thermal stability under high geothermal temperatures. These results indicate that relatively thin polymer coatings may provide effective thermal insulation without significantly impeding drilling fluid flow [146].

**Figure 7:**
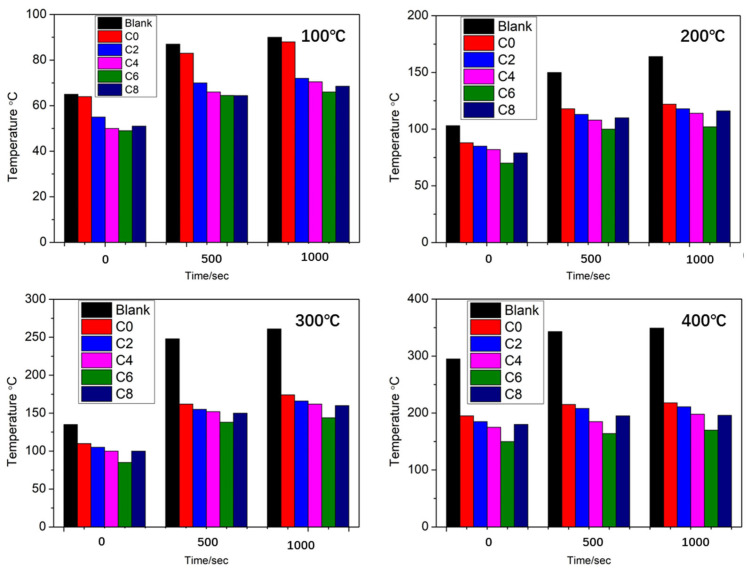
Thermal insulation temperature difference as a function of time at different environmental temperatures of 100 °C, 200 °C, 300 °C, and 400 °C, re-plotted based on data from [150].

Aerogel-based polymer composites represent a significant advancement in the thermal insulation of TIDPs, attracting considerable attention due to their ultra-low thermal conductivity, typically ranging from 0.01 to 0.02 W·m^−1^·K^−1^ [147]. These materials possess extremely high porosity, which significantly suppresses heat transfer by conduction, making them particularly effective for deep geothermal and high-temperature high-pressure (HTHP) drilling applications. Experimental and modeling studies have shown that aerogel-lined TIDPs can reduce drilling fluid temperature losses by up to 25% even under HTHP conditions, thereby maintaining fluid thermal stability and reducing energy input requirements [148]. To overcome the inherent brittleness of aerogels under downhole conditions, the development of polymer–aerogel hybrid composites has been proposed. These hybrids combine the excellent insulating performance of aerogels with enhanced mechanical robustness, improving their suitability for drilling applications [149]. For example, silica aerogel–based coatings exhibited a minimum thermal conductivity of 0.05748 W·m^−1^·K^−1^ at an optimal aerogel loading of 6 g, and at 4 g the thermal conductivity increased to 0.10628 W·m^−1^·K^−1^, while the thermal conductivity of the aerogel filler itself was 0.0232 W·m^−1^·K^−1^ [150]. At an average hot-face temperature of 351 °C, the coating maintained a back-face temperature of 188 °C, resulting in a temperature difference (ΔT) of 163 °C, compared to 134 °C in the absence of aerogel. The ΔT increased progressively between 100 and 400 °C (from 24 to 163 °C for C6 and 18 to 134 °C for C0), confirming the effectiveness of aerogel incorporation, as illustrated in Figure 7. Heat treatment at 100 °C and 200 °C had no significant impact on thermal performance; however, excessive aerogel loading led to particle agglomeration and a slight reduction in insulation efficiency.

**Figure 8:**
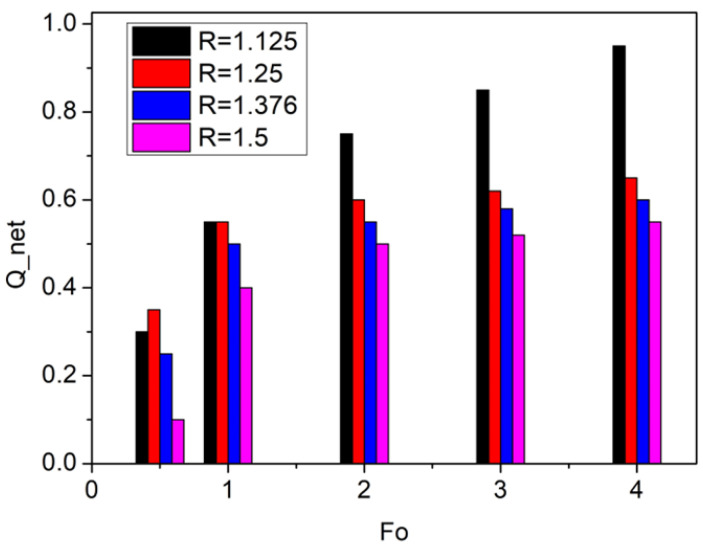
The effect of the PCM layer thickness (R) on net heat loss ratio (Q_net) at different Fourier numbers (F_o_), re-plotted based on data from [158].

Vacuum insulation has also been demonstrated to significantly enhance the thermal performance of TIDPs, particularly in deepwater and HTHP drilling environments. By creating an evacuated annular space within dual-wall drill pipes, both conductive and convective heat transfer mechanisms are effectively suppressed. Under high-vacuum conditions, the thermal conductivity can be reduced to values as low as 0.008 W·m^−1^·K^−1^. To achieve this level of thermal conductivity, an internal gas pressure of approximately 200 mbar (2.0 × 10^4^ Pa ≈ 0.20 atm) is typically required. However, as the vacuum is partially lost, the number of gas molecules within the insulation layer increases, which enhances gas-phase conduction and raises the effective thermal conductivity. Consequently, the thermal conductivity gradually increases from very low values under high-vacuum conditions to higher values as the pressure rises toward partial-vacuum conditions [151]. This performance surpasses that of both internal and external polymer coatings, confirming the superior capability of vacuum-insulated TIDPs to maintain thermal safety during HTHP drilling operations. In addition, the incorporation of reflective metallic layers within the vacuum gap can further reduce radiative heat transfer, with the multilayer configuration functioning as an advanced vacuum insulation system [152]. Among existing insulation technologies, vacuum-based TIDPs are regarded as the most reliable solution for geothermal and HTHP drilling applications. Their ability to combine ultra-low thermal conductivity with effective radiative shielding makes them superior to traditional polymer coatings and many advanced composite systems. However, maintaining long-term vacuum integrity under extreme downhole pressures remains a significant technical challenge [153]. For instance, Wu et al. demonstrated that dual-wall vacuum-insulated drill pipes could substantially reduce bottom-hole circulating temperatures (BHCT) to approximately 59 °C in vertical wells and 65 °C in horizontal wells [154]. This performance clearly outperforms both internal and external polymer coatings, highlighting the effectiveness of vacuum insulation in preserving thermal safety during HTHP drilling operations, as shown in Figure 8. Furthermore, the thermal efficiency of vacuum insulation systems can be further enhanced by integrating reflective metallic layers.

Polymer–phase change material (PCM) systems have been proposed to mitigate transient temperature fluctuations in geothermal and high-temperature high-pressure (HTHP) wells [155]. PCMs used for the thermal insulation typically exhibit latent heat capacities ranging from about 100 to 400 kJ/kg, depending on composition. CMS absorb and release latent heat during phase transitions, typically between solid and liquid states, thereby providing thermal buffering against sudden downhole temperature variations. Incorporating PCMs into polymer matrices or coating systems can effectively counter thermal shocks that may otherwise damage equipment or interrupt drilling operations [156]. This heat-buffering capability makes PCM-based TIDPs particularly advantageous in drilling operations involving frequent circulation stoppages. During these stoppages, PCMs can reduce the temperature by approximately 8–15 °C, depending on their composition [157]. For example, Alawadhi investigated the use of a PCM layer positioned on the inner surface of pipe insulation to enhance thermal efficiency [158]. A one-dimensional finite element model with convective boundary conditions was employed to analyze the effects of PCM type, layer thickness, and thermal cycling behavior. The results demonstrated that incorporating a PCM layer of appropriate thickness significantly reduced heat loss compared to conventional insulation, with higher PCM volume fractions yielding improved thermal performance, as shown in Figure 8. Moreover, the PCM-enhanced insulation exhibited stable long-term performance and showed minimal sensitivity to variations in thermal cycling regimes.

Despite the progress achieved with polymer-based, aerogel-based, and PCM-enhanced TIDPs, several limitations remain. Polymer coatings exhibit relatively moderate thermal conductivity, which restricts their effectiveness under extreme thermal gradients. Although aerogel-based systems offer exceptional insulation efficiency [146], they suffer from challenges related to mechanical durability and moisture sensitivity, which can adversely affect long-term performance [159]. PCM-based systems require precise matching of phase-change temperatures with downhole operating conditions and may experience performance degradation over extended thermal cycling periods [160]. Future research is therefore expected to focus on hybrid insulation systems that integrate aerogels, PCMs, vacuum layers, and reflective polymer composites into multifunctional coatings with enhanced mechanical strength, corrosion resistance, and wear resistance. Such advanced systems are expected to provide more durable and reliable solutions for deeper and hotter wells, enabling safer and more energy-efficient drilling operations. Overall, evaluations of TIDP thermal performance indicate that no single insulation technology is universally superior. In HPHT and geothermal applications, hybrid approaches that combine PCM, vacuum insulation, aerogel, and polymer layers appear to offer the optimal balance between insulation efficiency, structural integrity, and operational feasibility.

### 6.2. Mechanical Performance

The mechanical properties of TIDPs are critical, particularly under HPHT and deep-water drilling conditions [161,162,163]. During operation, TIDPs are subjected to extreme tensile, compressive, and torsional loads; therefore, structural integrity must be maintained even with the incorporation of insulating layers. Adequate mechanical strength is essential to ensure drilling safety, minimize downhole failures, and extend service life. Key indicators of mechanical performance include tensile strength, compressive strength, collapse resistance, fatigue endurance, and abrasion resistance [164,165,166]. Standard laboratory evaluations typically involve simulated downhole loading conditions, including tensile and compressive load testing, fatigue cycling, collapse pressure testing, and abrasion resistance measurements. These laboratory results are often validated through field testing, such as assessments of coating adhesion, wear resistance, and overall performance under operational conditions. In recent years, polymer-based, vacuum-based, and PCM-based TIDPs have been increasingly applied not only for thermal insulation but also as mechanical protection layers [167,168,169,170]. The following section provides a comprehensive discussion of the mechanical performance of these TIDP systems.

**Figure 9:**
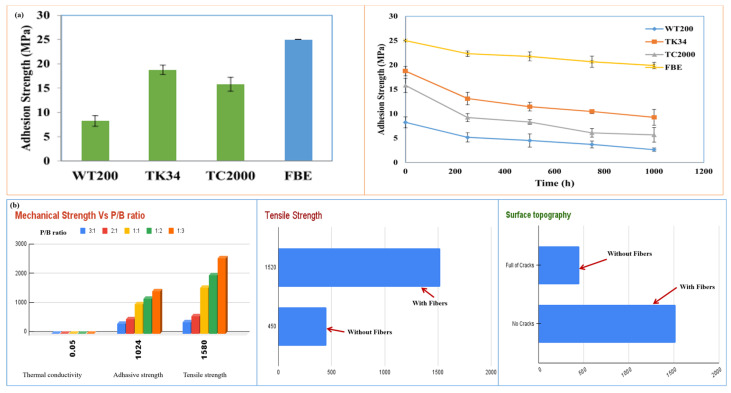

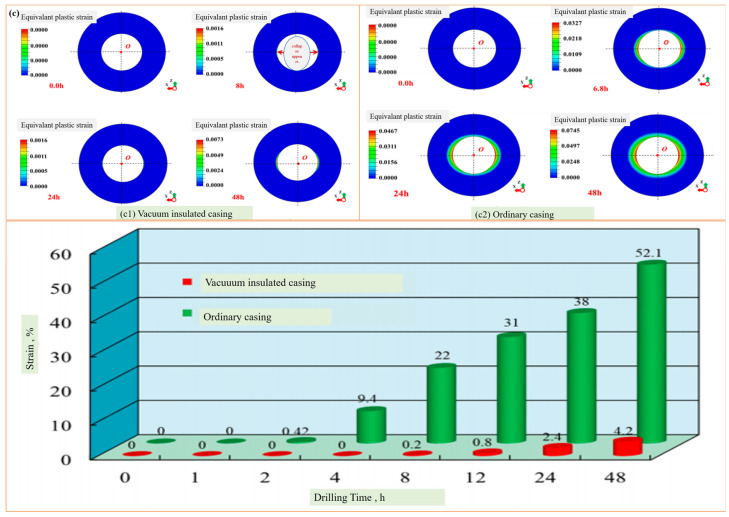
Mechanical performance of TIDPs: (**a**) Adhesion test results of different type of coated drill pipe and adhesion strength of coatings after a salt spray test [175], (**b**) Thermal conductivity and adhesive strength of the coating with various P/B ratios and tensile strength and surface topography with fiber and without fibers re-plotted based on data from [95], (**c**) Distribution of the final yield region around well bore for both cases and Evolution curve of parameter δ [101].

Polymer coatings significantly enhance the mechanical durability of TIDPs [171,172,173]. Drill pipes are typically manufactured from high-strength steels, such as V150 grade steel (yield strength exceeding 150,000 psi), and polymer coatings serve to protect and reinforce these substrates. Lach et al. reported that polymer coatings improve adhesion to steel surfaces and alleviate stress concentrations, thereby reducing the risk of mechanical failure under tensile and bending loads [174]. Field tests conducted by Vetsak demonstrated that epoxy-coated insulated drill pipes retained more than 96% coating integrity under extreme downhole conditions, even when exposed to severe shear, abrasion, and impact forces [9]. Furthermore, composite polymer coatings reinforced with ceramic fillers exhibit enhanced abrasion resistance and microhardness. In 2021, Bigdeli conducted a comparative study on the mechanical properties of various epoxy-based coatings applied to drill pipes, including fusion-bonded epoxy (FBE) and three commercially available liquid epoxies (TC2000, TK34, and WT200). FBE exhibited superior adhesion strength (>25 MPa) compared with TK34 (~18 MPa), TC2000 (~15 MPa), and WT200 (~8 MPa), as well as high hardness (~88 Shore), comparable to TK34 (~86 Shore) and exceeding the other formulations (~83 Shore) [175]. Impact testing revealed that FBE and TK34 coatings did not exhibit cracking at higher impact energies (20 J), whereas TC2000 and WT200 failed at lower energies (~16 J and ~13 J, respectively). Abrasion testing further confirmed the superior performance of FBE, which showed the lowest mass loss (~45 mg), followed by TK34 (~67 mg), as illustrated in Figure 9a. These results clearly demonstrate the advantages of polymer coatings, particularly FBE, in maintaining mechanical integrity under the extreme conditions encountered by TIDPs [175].

Aerogels, which are well known for their exceptionally low thermal conductivity, have also been increasingly investigated with an emphasis on enhancing mechanical performance [176,177,178]. Their incorporation into composite TIDP systems can improve compressive strength while maintaining a low overall density. Teng [179] and Dang et al. [161] further reported improved fatigue tolerance up to 30% compared with steel specimens coated with standard epoxy because polymer aerogel composite insulation systems are capable of absorbing mechanical shocks and reducing stress concentrations along the specimens surface. However, these tests were performed on coated steel specimens under simplified mechanical loading conditions and did not incorporate factors such as internal pressure differentials, thermal cycling representative of downhole temperature gradients, or exposure to drilling fluids. In addition, the reported fatigue improvements were obtained under controlled laboratory conditions rather than under standardized drill pipe fatigue testing protocols, such as API RP 7G or ISO 16135. Therefore, the direct applicability of these results to the operational fatigue performance of drill pipes remains uncertain. In 2022, Li et al. observed that aerogel-based coatings retained their structural integrity at temperatures exceeding 200 °C, at which many conventional metallic protective coatings typically degrade [180]. This combination of high thermal performance and enhanced fatigue resistance makes aerogel–polymer hybrid systems particularly attractive for ultra-deep drilling applications. For instance, formulation optimization has been shown to significantly improve the mechanical performance of silica-aerogel-based thermal insulation coatings. The incorporation of glass and ceramic fibers effectively reinforced the composite against crack propagation, increasing tensile strength from 450 kPa (without fibers) to 1520 kPa while simultaneously reducing surface cracking. Although the addition of silica aerogel enhanced thermal insulation, it reduced adhesive strength due to its highly porous structure. An optimal 1:1 ratio of silica aerogel to hollow glass microspheres achieved the best balance, yielding a thermal conductivity of 0.050 W·m^−1^·K^−1^ and an adhesive strength of 1024 kPa. Similarly, Xue et al. demonstrated that a pigment-to-binder (P/B) ratio of 1:1 provided optimal adhesion and insulation performance, with particle size and fracture behavior indicating a strong correlation between mechanical strength and adhesion efficiency (Figure 9b) [95]. Future work should investigate the fatigue performance of aerogel-based TIDP insulation systems using standardize drill pipe fatigue testing procedures under realistic drilling conditions.

Vacuum-based TIDPs, typically designed with dual-wall structures, exhibit outstanding mechanical and thermal performance. Vacuum-insulated pipes have demonstrated the ability to withstand severe external conditions at depths exceeding 3000 m, where the risk of structural failure is substantial [181,182,183]. Maintaining vacuum integrity requires strong adhesion between the steel substrate and the insulation layers. Recent advancements have therefore incorporated vacuum gaps in combination with hybrid coatings to enhance structural stability and reduce the risk of delamination under rapid pressure fluctuations [184,185]. In addition, reflective metallic coatings contribute to mechanical durability by providing an outer layer with enhanced wear resistance [186,187]. Consequently, vacuum-based TIDPs offer an effective combination of mechanical robustness and thermal insulation, particularly for HPHT drilling applications. For example, a simulation study investigated the influence of vacuum loss in insulated casings on wellbore stability in permafrost environments, providing valuable insights applicable to vacuum-based TIDP systems under similar mechanical loading conditions [101,188]. The results showed that vacuum-insulated casings reduced the radius of the yield zone by 63% compared with conventional casings. No yielding was observed during the first 48 h of drilling with vacuum insulation, whereas significant yielding occurred after only 1.83 h in conventional casings. Moreover, the yield parameter decreased from 52.1% in conventional designs to just 4.2% in vacuum-insulated casings, clearly demonstrating the enhanced structural resilience provided by vacuum insulation in response to thermally induced stresses (Figure 9c) [101].

PCM-based thermal interface layers can enhance mechanical integrity through multilayer structural reinforcement. According to Lee et al., PCM–polymer composites exhibit improved resistance to thermal shock, thereby indirectly enhancing fatigue performance under cyclic loading conditions [189,190]. The interfacial bonding between encapsulated phase change materials and polymer matrices contributes to structural stability even under high compressive loads. This design mitigates crack propagation and minimizes the risk of delamination, rendering PCM-based TIDPs mechanically viable under fluctuating HPHT conditions [191,192]. However, the long-term mechanical stability of these systems under repeated thermal cycling remains a significant research challenge. For example, Sacchet et al. investigated PCMs embedded in high-density polyethylene (HDPE) to produce shape-stabilized composites suitable for TIDP insulation applications. The PCM/HDPE composites exhibited appreciable tensile strength, suggesting that PCM incorporation promotes non-catastrophic mechanical failure behavior rather than abrupt fracture [193,194]. Elastic modulus and hardness were evaluated at room temperature and after complete PCM melting to assess structural performance under operational conditions. While PCM incorporation significantly enhanced thermal energy storage capacity, it partially compromised mechanical properties, indicating that a trade-off between thermal efficiency and mechanical strength is often unavoidable [195].

Despite these advances, optimizing the mechanical performance and operational reliability of the TIDPs remains challenging. Although polymer coatings offer high strength and good adhesion, prolonged exposure to elevated temperatures and aggressive chemical environments can lead to material degradation and loss of mechanical integrity [196,197,198]. Aerogels, while excellent thermal insulators, require mechanical reinforcement due to their inherent brittleness. Vacuum-based systems provide superior collapse resistance and cyclic load endurance; however, maintaining long-term vacuum integrity under downhole operating conditions remains technically challenging [190]. PCM-based systems show promise in mitigating thermal shock and moderating temperature fluctuations, but their long-term cyclic stability requires further improvement. In addition to mechanical durability, potential failure modes of insulated drill pipe systems must also be considered from an operational safety perspective. For example sudden loss of vacuum in a vacuum-insulated drill pipe may significantly increase radial heat transfer to the circulating drilling fluid, potentially causing localized thermal expansion and pressure fluctuations within the wellbore [75,199,200]. Such rapid thermal changes could influence equivalent circulating density (ECD) and may contribute to well control challenges under certain drilling conditions [41,199]. Similarly, mechanical damage to encapsulated phase change material (PCM) layers could lead to the leakage of PCM components into the drilling fluid, potentially altering fluid rheology and affecting cuttings transport or pressure management [201]. Although these scenarios remain relatively rare and are largely dependent on insulation system design and operational conditions, they highlight the importance of incorporating reliability assessment, robust sealing systems, and monitoring strategies when deploying TIDPs in high pressure high temperature (HPHT) wells. Future developments are therefore expected to focus on hybrid multilayer architectures that integrate polymer coatings, aerogels, syntactic foams, phase change materials, and vacuum gaps into multifunctional composite systems [202,203,204]. These next-generation TIDPs are anticipated to combine high tensile strength, enhanced fatigue resistance, chemical durability, and superior thermal insulation within a single structural design, thereby improving drilling safety and cost-effectiveness in deep and ultra-deep wells. Overall, these advancements indicate that while vacuum- and polymer-based systems significantly enhance mechanical strength, PCM- and aerogel-based materials offer substantial opportunities for hybrid reinforcement. For next-generation TIDPs, the primary engineering challenge remains achieving an optimal balance among fatigue resistance, flexibility, adhesion, and long-term durability under extreme downhole conditions. Therefore, future TIDP development should integrate both thermal–mechanical optimization and operational safety evaluation to ensure reliable deployment in deep and ultra deep drilling environments.

### 6.3. Corrosion Resistance

TIDPs must exhibit high corrosion resistance, particularly in HPHT and deep-water drilling applications, where long-term exposure to aggressive environments can compromise pipe integrity and durability [10,11]. Production environments are commonly associated with corrosive fluids, including hydrogen sulfide (H_2_S), carbon dioxide (CO_2_), chlorides, and acidic brines, which can induce pitting corrosion, sulfide stress cracking (SSC), and general chemical degradation. Corrosion resistance refers to the ability of TIDPs to withstand chemically aggressive environments while maintaining their mechanical and thermal performance. Common evaluation methods include electrochemical impedance spectroscopy (EIS), salt spray testing, and immersion tests in corrosive media such as 3 wt% NaCl or 5 wt% H_2_SO_4_, which are used to assess coating delamination, blister formation, and mass loss [167,205]. Laboratory results are typically validated through field testing in sour hydrocarbon wells to assess degradation under realistic operating conditions. In recent years, polymer-based, vacuum-based, and PCM-based TIDPs have been increasingly applied as corrosion-protection systems. The corrosion performance of these TIDPs is comprehensively discussed in the following section.

**Figure 10:**
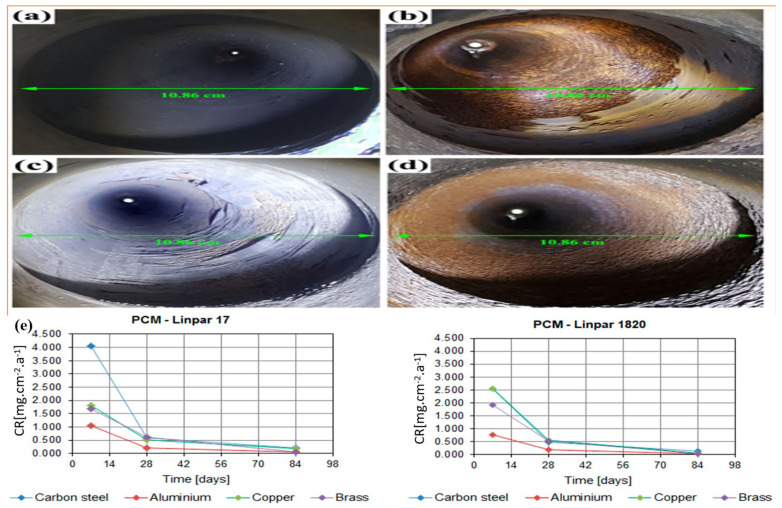
Corrosion tests images of epoxy coatings for drilling pipes: (**a**) FBE coating, (**b**–**d**) TC2000 coating [175], (**e**) Dependence of corrosion rate of the tested different metals on the time of immersion in organic PCMs: (left) Linpar 17; (right) Linpar 1820 [217].

Polymer coatings are widely used in TIDPs due to their chemical stability, tunable properties, and strong adhesion to steel substrates. High-performance polymers, including epoxy resins, polyimides, and fluoropolymers, serve as effective protective barriers against corrosive drilling fluids. For instance, polyether ether ketone (PEEK) liners have demonstrated excellent chemical resistance in strongly acidic environments (pH < 3) at temperatures exceeding 250 °C, with minimal degradation [206,207,208]. Feng et al. showed that epoxy coatings incorporating nano-silica or alumina exhibited reduced diffusion rates of corrosive agents while maintaining adhesion after hydrothermal aging, indicating improved long-term corrosion resistance [209,210,211]. Fluoropolymer topcoats, such as polyvinylidene fluoride (PVDF), have also been applied in offshore and shallow-water drilling operations, where they provide low permeability to acids and hydrocarbons [212]. A comparative evaluation of fusion-bonded epoxy (FBE) and liquid epoxy coatings applied to ST37 steel drill pipes assessed service life, structural integrity, and corrosion resistance using salt spray exposure, acid immersion tests, and EIS measurements. The results demonstrated that FBE coatings exhibited the lowest corrosion rates across all testing conditions (Figure 10a–d). This superior performance was attributed to the higher coating density and reduced porosity of FBE, which effectively impeded the ingress of aggressive species to the steel substrate [175,213].

Incorporation of silica aerogels (SA) into polymer coatings further enhances corrosion resistance by sealing micro-pores and forming effective diffusion barriers that inhibit corrosive penetration [52,214]. He et al. reported a direct correlation between increasing SA content and improved corrosion resistance. Coatings containing 6 g of SA exhibited no delamination or blistering after prolonged immersion in 5 wt% H_2_SO_4_ and 3 wt% NaCl solutions [150]. This enhanced performance was attributed to the increased surface area and structural reinforcement provided by the aerogel network, which improved both chemical resistance and mechanical integrity. These findings demonstrate that aerogel-modified polymer coatings are well suited for HPHT drilling environments where conventional coatings often fail to meet long-term corrosion protection requirements.

Vacuum-based TIDPs have demonstrated indirectly enhanced corrosion resistance, primarily due to reduced temperature gradients and limited interaction between corrosive fluids and the load-bearing inner pipe. These systems are typically designed as dual-wall insulated pipes, in which the vacuum layer minimizes heat transfer and restricts fluid ingress, thereby mitigating thermally accelerated corrosion processes. PCM-based insulation systems are primarily intended for thermal buffering; however, they can also contribute to corrosion protection through multilayer composite designs. Łach et al. reported that incorporating phase change materials within polymer matrices reduces thermally induced stresses during cyclic heating and cooling, which are key contributors to crack initiation and coating failure [174]. By limiting temperature fluctuations, PCM-containing coatings suppress micro-crack formation, thereby reducing the exposure of the metallic substrate to corrosive drilling fluids [215]. In addition, PCM–polymer composites can be engineered with functional fillers, such as carbon nanotubes or ceramic particles, to improve barrier properties and enhance abrasion resistance [216]. Although research in this area is still at an early stage, PCM-enhanced systems show considerable potential for improving corrosion resistance under dynamic HPHT drilling conditions. For example, a study investigating the compatibility of PCMs with various metallic substrates highlighted the critical role of material selection in TIDP design. The results showed that certain alloys were susceptible to galvanic corrosion when in contact with specific PCMs, whereas aluminum alloys exhibited superior corrosion resistance, as illustrated in Figure 10e. These findings emphasize that appropriate substrate selection is essential for minimizing corrosion-related risks in PCM-based TIDPs [217].

Despite significant progress in corrosion mitigation, TIDPs still face challenges related to long-term durability under severe downhole conditions. Polymer coatings may degrade over extended exposure to high temperatures and aggressive chemical environments [218]. Although aerogels improve barrier performance, their inherent brittleness necessitates reinforcement for mechanical reliability [219]. Vacuum-based systems offer indirect corrosion protection benefits, but maintaining long-term vacuum integrity during extended drilling operations remains challenging [220]. Similarly, while PCM-based coatings are promising, their long-term cyclic stability and compatibility with drilling pipe materials require further validation [221]. Future research is therefore expected to focus on multifunctional coating systems that integrate corrosion resistance, thermal insulation, wear resistance, and self-healing capabilities. Bouquerel et al. suggested that next-generation TIDPs are likely to incorporate nano-engineered polymers, aerogels, PCMs, and vacuum technologies to provide comprehensive protection in HPHT and deepwater drilling environments [222]. Overall, corrosion resistance in TIDPs is governed by a balance between coating compactness, adhesion strength, and chemical compatibility with HPHT drilling fluids. Emerging self-healing coatings and advanced nanocomposite technologies offer promising pathways to enhance long-term durability without compromising mechanical or thermal performance.

### 6.4. Operational Longevity and Economic Viability

The operational lifespan of TIDPs depends on their ability to maintain mechanical integrity, thermal insulation, and protective functionality over extended drilling periods under HPHT conditions, as well as in abrasive and corrosive environments. Economic viability is achieved by balancing the initial capital investment against long-term energy savings, reduced maintenance requirements, and minimized operational downtime. Standard durability evaluation methods, including rapid thermal cycling, abrasion and wear resistance testing, and long-term corrosion immersion tests, are commonly employed to simulate material degradation and predict long-term performance. In addition, life-cycle assessment (LCA) models, combined with field trials conducted in geothermal and deepwater wells, are widely used to evaluate the overall cost efficiency and performance of TIDP systems [223,224]. In recent years, polymer-based, vacuum-based, and PCM-based TIDPs have been increasingly deployed to enhance operational longevity and economic viability. The following section comprehensively discusses the durability and cost-effectiveness of these TIDP technologies.

**Figure 11:**
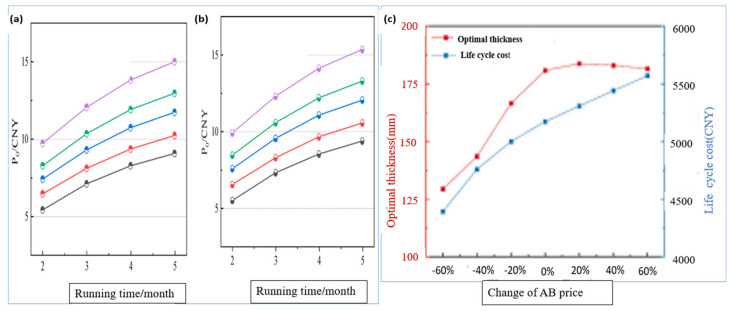
Total cost of insulation thickness per unit length of different pipeline models: (**a**) rock wool and (**b**) glass wool [227]. (**c**) Influence of the aerogel price on optimal thickness and life cycle cost (AB + GW) [231].

Polymer coatings play a critical role in improving the durability and cost efficiency of TIDPs. High-performance polymers such as PEEK, polyimides, and epoxy composites exhibit strong resistance to thermal degradation and chemical attack; however, they remain more susceptible than ceramic coatings under extremely harsh downhole conditions. Field studies have shown that TIDPs with polymer coatings may experience insulation performance losses of up to 20% within two years, whereas ceramic-coated systems exhibited losses of less than 5% in geothermal wells operating at approximately 300 °C [225]. To address this limitation, advanced formulations—such as epoxy coatings reinforced with silica nanoparticles or incorporating self-healing functionalities—have demonstrated improved resistance to micro-cracking and delamination, thereby extending service life to levels comparable to ceramic-based systems [226]. From an economic perspective, polymer-based insulation solutions offer advantages including lower maintenance costs, flexible application processes, and compatibility with additive manufacturing techniques, all of which contribute to reduced downtime and material waste during production [138]. For instance, recent advances in polymer technology—particularly the incorporation of silica nanoparticles and carbon nanotubes—have significantly enhanced thermal stability and mechanical strength, leading to longer service lifetimes and reduced replacement frequency for TIDPs [69]. Although polymer-coated TIDPs require higher initial capital investment than conventional drill pipes, life-cycle cost (LCC) analyses indicate that long-term operational efficiencies can offset these upfront costs. Xu et al. [227] demonstrated that the key economic determinants for optimal insulation design in drilling pipelines include pipe diameter, operating duration, drilling fluid temperature, and energy costs. Pipelines equipped with electrical heat tracing require substantially thicker insulation layers than non-traced systems, resulting in higher overall costs. Rock wool insulation, while more expensive per unit mass than glass wool, offers lower thermal conductivity, enabling thinner insulation layers and potentially reducing total system costs. The proposed model provides a scientifically grounded and economically viable framework for insulation design, facilitating heat loss reduction and improved cost efficiency in drilling operations, as illustrated in Figure 11a,b.

Silica aerogels (SAs) have been developed as lightweight, highly insulating materials that significantly enhance both the durability and cost-effectiveness of thermally insulated drill pipes (TIDPs). Heat loss is substantially reduced by the highly porous structure of aerogels, which forms effective air barriers that impede heat transfer and limit the penetration of corrosive species. Akbarzade et al. demonstrated that polymer–aerogel composite coatings maintained structural integrity and chemical resistance during prolonged exposure to aggressive chemical environments, with high-loading aerogel formulations outperforming conventional polymer coatings [228]. Aerogel-based coatings thus provide economic value by reducing energy consumption associated with heating drilling fluids and extending pipe service life in HPHT applications, thereby lowering replacement and maintenance costs. Although aerogel systems typically involve higher initial production costs, their superior thermal insulation and corrosion resistance can lead to substantial life-cycle cost savings in deepwater and geothermal drilling operations [229]. Aerogel insulation layers are particularly valued for their high thermal stability and ultralow thermal conductivity, and studies have shown that aerogel blankets retain their insulating performance over extended service periods [93]. In industrial steam systems, aerogel insulation has demonstrated the ability to withstand high temperatures and harsh environmental conditions, thereby enhancing system longevity. Life-cycle cost analyses further indicate that aerogel-based composite insulation can reduce total costs and improve energy efficiency in applications such as district heating networks and high-temperature piping systems [230]. However, the temperature resistance of insulation materials must be carefully considered during design; otherwise, pipeline life-cycle costs may be underestimated and safety margins inadequately addressed. While composite insulation systems are often thermally and economically superior to single-layer designs, they typically require greater total thickness. Aerogel blankets (ABs) offer excellent insulation at reduced thickness but at relatively high cost; combining aerogel blankets with glass wool (AB + GW) provides an optimal balance between performance and cost. Studies have shown that the AB + GW configuration achieves the lowest life-cycle cost, shortest payback period, and highest thermal efficiency across a range of steam temperatures and pipe diameters, with a temperature resistance of up to 300 °C. The proposed model thus serves as a practical and scientifically grounded tool for designing safe, cost-effective insulation solutions for high-temperature district heating and industrial piping systems, as illustrated in Figure 11c [231].

Dual-wall TIDP designs incorporating evacuated annular gaps based on vacuum insulation concepts offer significantly enhanced thermal insulation and extended service life. These systems reduce thermal stresses induced by steep temperature gradients, which are often responsible for crack initiation, corrosion onset, and insulation failure [232]. Gubbels and Santi [233] reported that vacuum-insulated TIDPs maintained structural and chemical integrity throughout prolonged drilling campaigns, exhibiting minimal degradation compared with conventional insulation systems. In addition to long-term energy savings, vacuum-based designs enable effective control of downhole fluid temperatures and reduce reliance on auxiliary heating systems [234]. Although fabrication costs are relatively high, their extended service life and reduced maintenance requirements make vacuum-insulated TIDPs economically attractive for multi-well operations, particularly in ultra-deepwater and HPHT environments. The longevity of vacuum insulation systems is influenced by factors such as internal gas pressure evolution, material degradation, and external environmental conditions [235]. Studies on commercially available vacuum insulation panels (VIPs) indicate that although thermal conductivity may increase over time due to material aging, these panels continue to provide effective insulation over extended periods [236]. Accelerated aging tests conducted under high-temperature and high-humidity conditions are commonly used to predict long-term performance and guide the design of vacuum-insulated TIDPs capable of meeting stringent downhole durability requirements [237]. Economic feasibility analyses indicate that vacuum insulation systems are highly sensitive to material costs, manufacturing technologies, and anticipated energy savings. Life-cycle assessments have shown that selecting low-cost core materials and optimizing fabrication processes can significantly reduce costs without compromising performance [238]. Although direct economic data for TIDPs remain limited, studies from building and industrial insulation applications demonstrate that VIPs can substantially reduce heat losses and enhance energy efficiency, suggesting strong potential for lowering long-term operational costs in downhole drilling applications [239].

PCM-based insulation represents a dynamic thermal management strategy that mitigates temperature fluctuations, thereby reducing thermal stress and extending the service life of TIDP. Studies have shown that PCM–polymer composites effectively regulate temperature variations and suppress crack formation, thereby minimizing degradation of insulating performance under repeated thermal and mechanical loading [240]. The incorporation of functional fillers, such as carbon nanotubes and ceramic particles, further enhances abrasion resistance and barrier properties [241]. From an operational perspective, PCM systems can reduce overall costs by stabilizing drilling fluid and downhole sensor temperatures, which in turn decreases non-productive time and reduces the frequency of pipe replacement. Although PCM-based coatings are still at an early stage of development and are largely supported by model-based analyses, experimental studies have demonstrated their potential. For instance, Osibodu and Adeyinka reported that incorporating PCMs into concrete improved thermal performance but reduced mechanical strength at higher PCM loadings, underscoring the importance of careful PCM selection and encapsulation strategies [242]. Cui et al. experimentally demonstrated that PCM integration effectively dampens temperature fluctuations, thereby enhancing thermal stability and extending system durability [243]. Beyond drilling applications, PCM integration has been widely recognized as economically advantageous in energy storage systems [244]. Studies on PCM-assisted air-conditioning systems have reported significant reductions in energy consumption and electricity costs by minimizing active heating and cooling requirements, supporting the long-term cost-effectiveness and sustainability of PCM-based systems [245].

Despite these advances, the long-term operational sustainability and cost efficiency of current TIDP insulation technologies remain constrained. While polymer coatings are relatively easy to repair and customize, their performance gradually degrades under prolonged HPHT exposure [246]. Aerogels exhibit exceptional thermal resistance but are inherently brittle and require mechanical reinforcement [247]. Vacuum-based insulation systems face challenges in maintaining vacuum integrity, particularly under mechanical impact or prolonged downhole vibration [248]. Although PCM-based coatings show considerable promise, further validation under realistic downhole conditions is required before widespread adoption [249]. Future development is therefore expected to focus on layered hybrid insulation systems that synergistically combine ceramics, polymers, aerogels, and phase change materials, along with self-healing functionalities, to achieve superior multifunctional performance. Luo and Li [176,250] emphasized the importance of incorporating advanced materials designed through predictive computational modeling, as well as surface modification techniques such as plasma treatment and chemical grafting, to enhance durability and reduce maintenance costs. Furthermore, as sustainability regulations become increasingly stringent, the recyclability of coated thermoplastics, such as polyether ether ketone (PEEK) and polyvinylidene fluoride (PVDF), is expected to emerge as a critical economic and environmental constraint [251]. Ultimately, the economic viability of TIDPs will depend on the successful integration of thermal insulation, mechanical robustness, and corrosion resistance within scalable and cost-effective manufacturing processes. To extend operational lifetimes and reduce total ownership costs, future research should prioritize the development of hybrid multilayer systems optimized through computational design and sustainable material selection. Although modern TIDP technologies have achieved substantial progress, persistent limitations remain; the following section, therefore, discusses these challenges and outlines potential pathways for further advancement.

## 7. Field Application of Thermally Insulated Drilling Pipes

TIDPs have been applied in the field as a downhole temperature-management technology to protect temperature-sensitive components (such as motors, MWD/LWD electronics, elastomers, and seals) and to maintain drilling-fluid performance in high-temperature geothermal and HPHT/HTHP wells. The first well-documented field application occurred in geothermal drilling. Finger and co-workers reported a controlled field test conducted in May 1999 in the Imperial Valley (southern California), involving a 3500-ft string of insulated drill pipe. In this test, circulating temperatures in the IDP were directly compared with those in conventional drill pipe over a range of flow rates and temperatures, and the temperatures measured in tools circulated within the insulated string were found to be lower than those observed with conventional drill pipe [252,253,254]. These geothermal tests were supported by geothermal drilling best-practices studies conducted by Sandia National Laboratories, which also identified IDP/insulation as a viable approach for maintaining lower fluid temperatures at depth when tool survivability is the limiting factor [15]. Subsequently, scale-up programs sponsored by DOE/NETL focused on transferring the technology from geothermal applications to deeper HPHT/HTHP drilling environments. These efforts addressed field-relevant issues such as inspection and handling, mechanical capability, and the trade-offs between thermal benefits, hydraulics, and operational complexity [88,255].

More recent field testing has been reported during Utah FORGE development drilling. Public drilling records document the successful use of insulated drill pipe and provide operational datasets, including mud-temperature logs and daily drilling reports, that can be used to advance thermal modeling and evaluate model performance [255]. In addition to these open datasets, recent field experience reported by Vetsak et al. (SPE/IADC) further complements the literature by providing thermal-model validation and quantified reductions in downhole tool temperatures achieved using a contemporary, purpose-built insulated drill pipe system. Their work also reports in-service experience related to coating and insulation wear, as well as field repairs between runs, indicating that deployment performance is governed not only by thermal resistance but also by abrasion and contact loading, outflow trajectory, and system maintainability [9].

Beyond geothermal applications, deep and ultra-deep wellbore cooling has attracted increasing attention, with numerous studies identifying TIDP as one of the most effective levers for controlling wellbore temperature during drilling, particularly when combined with surface cooling and optimized circulation parameters [256]. More recent studies further suggest that deployment strategy, such as full-string versus staged or partial placement of insulated joints, can strongly influence temperature control and cost effectiveness. Accordingly, selective use of insulation, rather than insulating the entire drill string, is supported in field practice, especially in sections of the string that most effectively minimize counter-current heat exchange [133,154,257].

Overall, practical field experience indicates that TIDP delivers the greatest benefits in operations where bottom-hole circulating temperature (BHCT) or thermal exposure is the dominant driver of operational risk. Effective implementation requires coordinated planning that accounts for (i) surface cooling capacity to manage potentially elevated return temperatures, (ii) hydraulic and pressure-loss impacts, and (iii) durability, inspection, and repair procedures under conditions involving contact, vibration, and thermal cycling.

## 8. Challenges and Future Perspectives

Table 3 summarizes the challenges and future perspective of the TIDPs. The reviewed studies indicate that the insufficient thermal and mechanical stability of polymer-based insulation coatings restricts the application of TIDPs under HPHT conditions. Most polymers begin to degrade at temperatures above approximately 350 °C, leading to molecular chain scission, oxidative degradation, and interfacial delamination, which reduce insulation performance and long-term durability. One of the primary research directions has therefore focused on enhancing thermal resistance through strategies such as improved cross-linking, as well as the incorporation of ceramic or nanomaterial reinforcements. However, manufacturing complexity remains a significant challenge, particularly for multilayer insulation systems. Hybrid manufacturing approaches, such as additive manufacturing for critical joints combined with conventional coating techniques for the main pipe body, may offer improved performance while maintaining cost efficiency. In addition, the lack of standardized HPHT testing protocols makes it difficult to reliably compare the performance of different materials. Future research should therefore focus on the development of thermally stable nanocomposite coatings, scalable manufacturing processes, and long-term performance evaluation under realistic drilling conditions.

## 9. Conclusions

TIDPs represent an important advancement for drilling operations in extreme environments such as deepwater, geothermal, and HPHT wells. Vacuum insulation provides the highest thermal efficiency among the studied technologies. Aerogel composites offer an excellent balance between low weight and strong insulation performance, making them particularly attractive for applications where both thermal management and structural considerations are important. Polymer coatings contribute by improving mechanical strength and corrosion resistance, thereby supporting the long-term durability of the system. PCM systems are effective for regulating transient temperature fluctuations, helping to stabilize thermal conditions during operation. However, several limitations remain. These include hydraulic penalties caused by the reduction in the inner diameter when insulation layers are added, the potential long-term degradation of the vacuum in vacuum-insulated systems, and concerns regarding the mechanical durability of aerogel materials under operational stresses. In addition, fatigue validation according to established drilling standards remains limited. Future research should therefore focus on the development of hybrid multilayer insulation systems and integrated thermo-hydraulic optimization to improve overall performance while addressing these technical challenges.

## Figures and Tables

**Table 3:** Current status, critical challenges and future perspectives of TIDPs.

General Direction	Current Status	Main Limitations	Recommendations
Polymer-based insulation coating	Moderate thermal conductivity (0.03–0.06 W/m·K); lightweight, flexible and easy to apply; widely used in industrial thermal protection coatings.	Limited high-temperature resistance (>200–300 °C), aging and oxidation, relatively higher thermal conductivity than advanced insulation materials	Development of nano-filled polymer composites(e.g., silica aerogel, ceramic nanoparticles); improved thermal stability polymers(polyimide, silicone-based); multilayer hybrid coatings.
Aerogel-based insulation	Ultra-low conductivity (0.015 W/m·K)	Brittleness, moisture sensitivity	Fiber-reinforced aerogel composites; surface functionalized
Vacuum-insulated system	Best thermal performance (0.008 W/m·K)	Long -term vacuum integrity	Advanced metallic sealing; getter materials
PCM integration	Effective transient thermal buffering	Leakage, thermal cycling degradation	Micro-encapsulation; high temp PCMs(>200 °C)
Additive manufacturing	Customized multilayer architectures	High cost scalability issues	Hybrid metal-ceramic AM; cost optimized DED
Hybrid multilayer TIDPs	Promising multifunctional behavior	Thermal–mechanical mismatch	Coupled thermo-mechanical modeling
Smart monitoring integration	Emerging concept	Sensor durability	Embedded fiber- optic DTS integration

## Data Availability

No new data were created or analyzed in this study.

## Nomenclature

**Symbol**	**Description**	**Unite**
k	Thermal Conductivity	W·m^−1^K^−1^
h	Convective heat transfer coefficient	W·m^−2^·K^−1^
q	Heat flux	W·m^−2^
σ	Stefan-Bolzmann constant	5.67 × 10^−8^ W.m^−2^·K^−4^
ε	Surface emissivity	-
R_e_	Reynolds number	-
ΔP	Pressure loss	Pa
ECD	Equivalent circulating density	ppg
TVD	True vertical depth	m
ΔT	Temperature difference	°C
R_total_	Total thermal resistance	m^2^·K/W
